# Safety outcomes of direct oral anticoagulants in older adults with atrial fibrillation: a systematic review and meta-analysis of (subgroup analyses from) randomized controlled trials

**DOI:** 10.1007/s11357-023-00825-2

**Published:** 2023-06-01

**Authors:** Katharina Doni, Stefanie Bühn, Alina Weise, Nina-Kristin Mann, Simone Hess, Andreas Sönnichsen, Susanna Salem, Dawid Pieper, Petra Thürmann, Tim Mathes

**Affiliations:** 1 Institute for Research in Operative Medicine, School of Medicine, Faculty of Health, Witten/Herdecke University, Ostmerheimer Str. 200, 51109 Cologne, Witten Germany; 2 Institute for Health Economics and Clinical Epidemiology of the University of Cologne, Gleueler Str. 176-178, 50935 Cologne, Germany; 3 Department of Clinical Pharmacology, School of Medicine, Faculty of Health, Witten/Herdecke University, Witten, Germany; 4 Institut für Wissensmanagement in der Medizin, Salzburg, Austria; 5 Department of Medical Statistics, University Medical Center Göttingen, Göttingen, Germany; 6 Philipp Klee-Institute for Clinical Pharmacology, Helios University Hospital Wuppertal, Wuppertal, Germany

**Keywords:** Older patients, Direct oral anticoagulants, Systematic review, Meta-analyses

## Abstract

Balancing stroke prevention and risk of bleeding in patients with atrial fibrillation (AF) is challenging. Direct oral anticoagulants (DOACs) are by now considered standard of care for treating patients with AF in international guidelines. Our objective was to assess the safety of long-term intake of DOACs in older adults with AF. We included RCTs in elderly (≥ 65 years) patients with AF. A systematic search in MEDLINE and EMBASE was performed on 19 April 2022. For determination of risk of bias, the RoB 2 tool was applied. We pooled outcomes using random-effects meta-analyses. The quality of evidence was assessed using GRADE. Eleven RCTs with a total of 63,374 patients were identified. Two RCTs compared apixaban with either warfarin or aspirin, four edoxaban with either placebo, aspirin, or vitamin K antagonists (VKAs), two dabigatran with warfarin and three rivaroxaban with warfarin. DOACs probably reduce mortality in elderly patients with AF (HR 0.89 95%CI 0.77 to 1.02). Low-dose DOACs likely reduce bleeding compared to VKAs (HR ranged from 0.47 to 1.01). For high-dose DOACS the risk of bleeding varied widely (HR ranged from 0.80 to 1.40). We found that low-dose DOACs probably decrease mortality in AF patients. Moreover, apixaban and probably edoxaban are associated with fewer major or clinically relevant bleeding (MCRB) events compared to VKAs. For dabigatran and rivaroxaban, the risk of MCRB varies depending on dose. Moreover, subgroup analyses indicate that in the very old (≥ 85) the risk for MCRB events might be increased when using DOACs.

Registration: PROSPERO: CRD42020187876.

**Supplementary Information:**

The online version contains supplementary material available at 10.1007/s11357-023-00825-2.

## Introduction

Balancing stroke prevention and risk of bleeding in patients with atrial fibrillation (AF) is challenging. Vitamin K antagonists (VKA) have been the main treatment for stroke prevention in AF patients in the past. However, the huge inter-individual variability of the clinical response, the necessity of monitoring the INR (International Normalized Ratio) and the quite unmanageable spectrum of food and drug interactions are major disadvantages of VKAs [1].

In the last decade, various direct oral anticoagulants (DOACs) entered the market which aimed to overcome these disadvantages. Randomized controlled trials (RCTs) on DOACs, namely rivaroxaban, apixaban, dabigatran, and edoxaban have shown a positive benefit-risk profile [2–9]. DOACs are by now considered the standard of care for treating patients with AF in international guidelines [10, 11].

Various patient related factors, in particular renal dysfunction, hepatic impairment and body weight can impact the pharmacokinetics of DOACs and consequently the risk for adverse events, such as major bleeding [12]. Studies under routine care conditions indicate that the real-world population differs from the one in RCTs with respect to these characteristics. These differences could have a significant impact on the benefit-risk ratio of DOACs [13]. Most conspicuous is that the population in real-world data-based studies is about ten years older than in RCTs [14–18]. As pharmacokinetics of DOACs are different in older adults when compared to younger patients, safety analysis of DOAC use in the elderly is of major interest [19].

A systematic review of RCTs and observational studies suggest superior effectiveness and similar safety of DOACs compared to VKAs and that apixaban probably has the best safety profile in geriatric patients (≥ 75 years) [20]. Likewise, recent observational studies based on real-world data suggest that DOACs are not associated with an increased bleeding risk compared to VKAs but results appear to depend on the specific DOAC and are heterogeneous across countries [14–18].

RCTs on safety outcomes of different DOACs and dosages in older adults would be desirable. However, considering the large sample size needed to adequately power such trials, it appears unlikely that such trials will be performed in the future. This is probably also the reason why existing evidence on the safety of DOACs in older adults mainly stems from observational study designs and indirect comparisons. These are generally at risk for confounding bias. Noticeably, confounding by indication for safety outcomes would mean that patients at higher risk for adverse events when using DOACs would have a lower chance to get a DOAC prescribed and consequently would mean a bias towards the null effect, i.e. would suggest no safety concerns [21].

Therefore, our objective was to assess the safety of long-term intake of DOACs in older adults with AF. Our analyses are based on data from RCTs or subgroup analyses from RCTs on older adults (≥ 65 years) to increase the applicability of the results to patients in routine care.

## Methods

We registered the protocol for this review in PROSPERO: CRD42020187876. All changes to the protocol are explicitly reported in the methods section.

This systematic review was performed according to the recommendations of the Cochrane Handbook for Systematic Reviews of Interventions [22] and follows the reporting recommendations of the updated Preferred Reporting Items for Systematic Reviews and Meta-Analyses (PRISMA) statement [23].

### Eligibility criteria

#### Participants

Eligible participants must be diagnosed with atrial fibrillation (AF) and above the age of 65 years. We operationalized the age criterion as follows:

-  ≥ 80% of the randomized population aged ≥ 65 years.
- Subgroup analysis reports on participants aged ≥ 65 years.

#### Intervention

The intervention group must be treated with any type of non-vitamin K antagonist oral anticoagulant. These include:

- apixaban
- dabigatran
- edoxaban
- rivaroxaban

We only included trials with long-term DOACs treatment, defined as a treatment duration of at least 12 months. This criterion was added during study selection because different from our expectation, we recognized that in some, mainly early phase RCTs DOACs treatment was very short, which is not comparable to routine care. Any dose or regimen was eligible. Trials on DOACs not approved in the European Union before 2020 (e.g., ximelagatran, darexaban, or letaxaban) were excluded.

As comparator, we accepted any active control such as conventional anticoagulation treatment, and no treatment, or placebo treatment. Furthermore, additional antithrombotic treatment in combined regimens (i.e. antiplatelet therapy in addition to warfarin) had to be the same in all groups, so that the groups only differed regarding DOACs treatment.

#### Outcomes

We prioritized all-cause mortality, all-cause hospitalization, and major or clinically relevant bleeding (MCRB) as primary outcomes (critical outcomes in GRADE). Secondary outcomes were any adverse event, discontinuation due to adverse events, renal failure, delirium, and falls (important outcomes). In addition, we extracted data on bleeding according to organ system classification.

We did not consider stroke or systemic embolism because we expected that the effectiveness of DOACs for reducing stroke is stable across age groups [24, 25] and consequently the subgroup effect of age would not shift the benefit-risk ratio.

#### Types of studies

Only RCTs or subgroup analyses of RCTs on the relevant age group were eligible.

#### Publication status

We only included trials published in English or German or with data available in an English language trial registry.

### Information sources

The identification of relevant literature comprised two stages.

First, we searched the reference lists of all systematic reviews included in a previous overview of reviews conducted by the research group of one member of our review team [24].

Second, we updated the electronic literature searches used in the aforementioned overview. For this purpose, MEDLINE, MEDLINE in Process, and Embase (all via Embase) were searched for studies published from 1st June 2014 (the last search of previous overview) onwards. We ran the last search on 19th April 2022.

In addition, we searched the reference lists of all included RCTs and systematic reviews on the same topic. Moreover, we searched ClinicalTrials.gov for ongoing and unpublished trials on 30 June 2020.

### Search strategy

The search strategy was prepared by an experienced information specialist in collaboration with clinical experts. The search terms used were e.g. *aged, atrial fibrillation,* or *anticoagulant agent*. The full search is presented in supplement I. The search was limited to English and German. In addition, we limited the search to articles and reviews (i.e., excluded conference abstracts) and excluded case reports, in vitro studies and animal experiments. The search included a search filter for the elderly, a modified generic search filter (in addition to specific terms such as bleeding or mortality) for adverse events and a validated search filter for RCTs [26–28]. The search strategy was reviewed by a second person using the PRESS-checklist and validated by checking if clearly eligible RCTs already known would have been identified [29].

### Selection process

Two reviewers independently screened the titles and abstracts of all records identified by the literature search. Next, full-text articles of potentially relevant reports were retrieved and assessed for compliance with the eligibility criteria by two reviewers independently. Disagreements between reviewers were resolved by discussion until consensus.

Multiple reports of the same RCT were merged, so that each trial is the unit of analysis. The study selection process was summarized in an updated PRISMA flow diagram [23].

### Data collection process

Descriptive data were extracted by one reviewer and checked for accuracy by a second reviewer. Two reviewers independently identified relevant outcome data by marking the section in the relevant source. Subsequently, one reviewer extracted the data, and a second reviewer checked for accuracy. All disagreements were resolved in discussions until consensus.

In case of missing data or inconsistent data on primary outcomes in different sources, we contacted the corresponding author by e-mail.

### Data items

Supplement II lists all items for which we extracted data.

We extracted data on outcomes for the last available follow-up, i.e. the longest observation period.

Supplemental to the outcome data, we extracted data on within study subgroup analyses. We only extracted data if the relevant subgroup analysis was pre-specified and a test of interaction was used to quantify the statistical certainty of the subgroup effect [30].

### Study risk of bias assessment

We assessed the risk of bias with the revised Cochrane risk-of-bias tool for RCTs (RoB 2 tool) [31]. The RoB 2 tool provides a framework for assessing the risk of bias for one particular outcome that is for each outcome separately.

### Effect measures

All considered outcomes were dichotomous. We extracted relative risk ratios from regression analyses (e.g., hazard ratios from a survival analysis) with 95% CIs. If these were not available (e.g., data from trial registries), we extracted raw data on events and number of participants for each group and calculated relative risks.

### Synthesis methods

#### Statistical synthesis method

We pooled data only if RCTs were sufficiently clinically and methodologically homogenous and the p-value of the statistical test for heterogeneity was > 0.05. To describe statistical heterogeneity, we calculated prediction intervals and I-square.

We pooled adverse event data separately for each comparator (VKAs, aspirin only, placebo) and dose because we assumed, they would have different risks, in particular for bleeding. We calculated systemic adverse events across AF patients (AF-only patients) and AF patients who had a percutaneous coronary intervention (AF-PCI patients), provided the patients were clinically comparable otherwise (e.g. renal function, comorbidity).

Mortality and hospitalization are composite outcomes, to be concrete measures that combine benefits (e.g. stroke reduction) and harms (e.g. bleeding). Therefore, for mortality and hospitalization we combined different comparators because we were interested in the net benefit of DOACs compared to all possible treatments that are applied in routine care. Moreover, we pooled mortality and hospitalization separately for AF and AF-PCI patients because the benefits of DOACs (e.g., stroke prevention) likely differ between AF and AF-PCI patients.

We derived the log standard errors, which are necessary for meta-analysis from the 95% confidence intervals (95% CIs). If more than one distinct subgroup for older adults was available (e.g. 65–74 years and ≥ 75 years), we pooled the results within one RCT using fixed effect meta-analysis. To combine different RCTs, we performed inverse variance random effects meta-analyses using the Hartung-Knapp method and the Paule–Mandel heterogeneity variance estimator [32, 33]. For outcomes for which only sparse data were available (event rate < 5%, zero event studies, less than four RCTs in meta-analysis) we planned to use beta-binomial regression models for sensitivity analyses [34, 35].

We used the R-Package Meta in R 9.4 for the meta-analyses [36]. In case of heterogeneity, we synthesized results across RCTs presenting range of effects of the point estimate of the relative risk ratio.

#### Subgroup analyses for exploring heterogeneity

We expected that our primary analyses would be mainly based on data from subgroup-analyses, and we had therefore not planned to perform subgroup analyses. However, in some meta-analyses there was statistically significant heterogeneity, and therefore we performed post-hoc subgroup analyses on study level according to agent.

#### Sensitivity analyses

We planned to perform a sensitivity analysis excluding RCTs at high risk of bias in the randomisation domain.

### Reporting bias assessment

We planned to assess publication bias by visual inspection of funnel plots for asymmetry, if at least 10 trials for each outcome were available.

We expected adverse events and mortality to be assessed in all RCTs. We considered RCTs/publications specifically on older adults in which mortality, overall adverse events, or discontinuation due to adverse events were not reported (and for which we got no information in response to author requests) susceptive for reporting bias. Bias in selection of the reported results within one trial is a domain of the RoB 2 tool (see above). In the RoB 2 assessment, we compared the list of outcomes reported in the protocols or methods section with the outcomes reported in the published paper.

### Certainty of evidence assessment

We rated the certainty of the body of evidence using the GRADE approach (Grading of Recommendations, Assessment, Development and Evaluation). In the GRADE system evidence from RCTs starts as “high-certainty” and the following criteria are applied for downgrading the certainty of evidence by one or two levels [37]:

- Risk of bias
- Imprecision
- Inconsistency
- Indirectness
- Publication bias

The rating of these criteria leads to four levels of the certainty of evidence for each of the prioritized outcomes [38]:

- High-certainty evidence: the review authors have a lot of confidence that the true effect is similar to the estimated effect.
- Moderate-certainty evidence: the review authors believe that the true effect is probably close to the estimated effect.
- Low-certainty evidence: the review authors believe that the true effect might be markedly different from the estimated effect.
- Very low-certainty evidence: the review authors believe that the true effect is probably markedly different from the estimated effect.

One reviewer judged the certainty of the evidence and a second reviewer verified the assessment. Disagreements were resolved by discussion until consensus.

The certainty of evidence and results are presented in 'Summary of Findings' (SoF) tables [39]. The SoF tables were prepared using GRADEpro GDT [40]. For estimating the absolute effect, we used absolute risks for the control group based on publications thought to be representative for routine care in Western countries [15, 16, 18]. If we could not find a suitable publication for one outcome, we used the risk of the comparator group of included RCTs.

To report the findings in consideration of the certainty of evidence, we used the standardized informative statements suggested by the GRADE working group [41].

The certainty of evidence is expressed with the following statements:

- High-certainty: reduces/increases outcome
- Moderate-certainty: “likely/probably” reduces/increases outcome
- Low-certainty: “may” reduce/increase outcome
- Very low-certainty: the evidence is uncertain

## Results

### Study selection

**Fig. 1:**
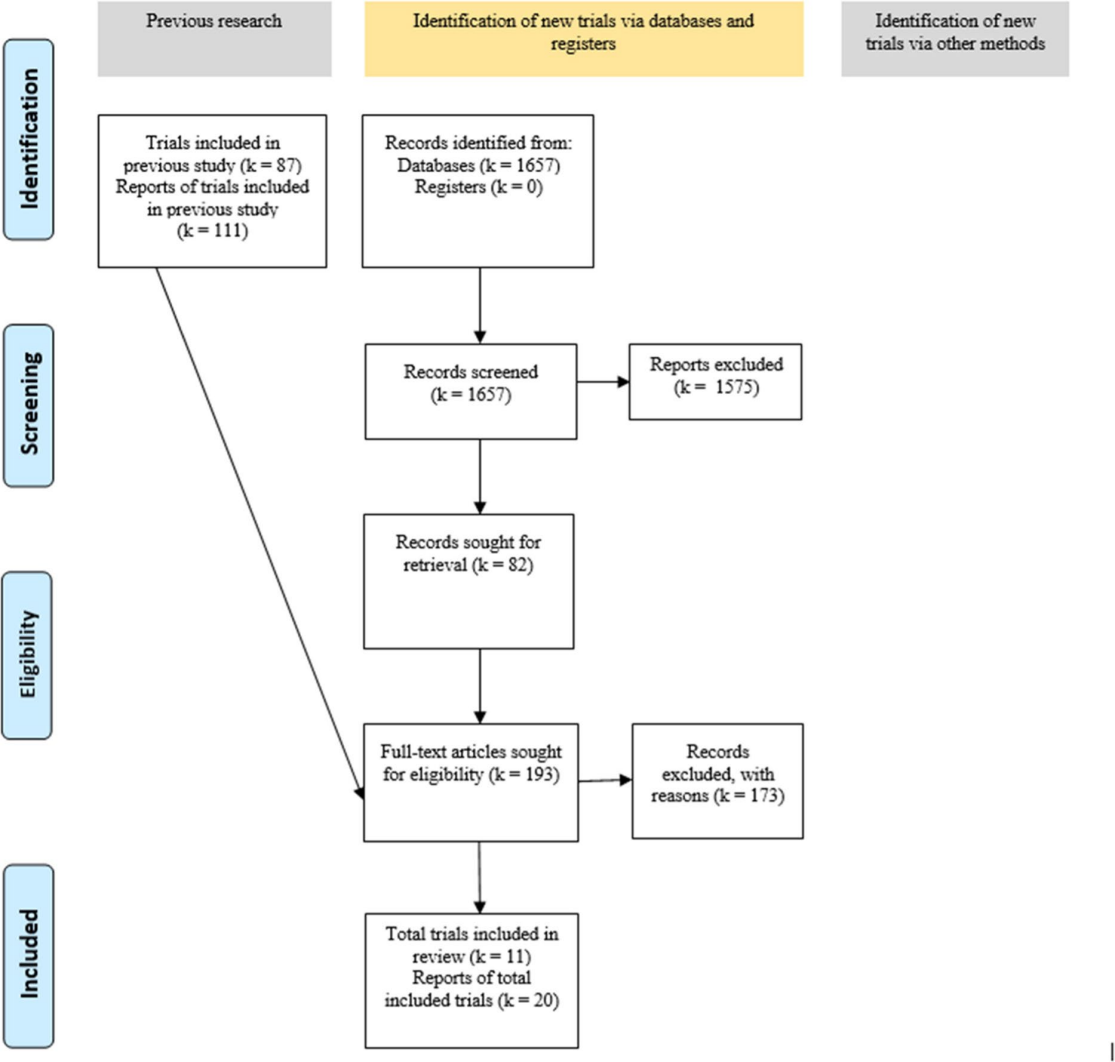
PRISMA 2020 flow diagram

Figure 1 shows the study selection according to the PRISMA statement [23]. The initial screening of publications included in the previously published overview [24] identified 87 potentially relevant RCTs (based on 111 trial reports) of which we screened full-text versions. The update electronic search provided a total of 1657 citations after duplicate removal. Titles/abstracts of these were screened and 82 potentially eligible study reports were identified. The screening of full-text publications yielded eleven RCTs (reported in 20 publications) which met all eligibility criteria [2–9, 42–44]. The search in ClinicalTrials.gov and the screening of reference lists of included RCTs and relevant systematic reviews did not lead to additional inclusions. A list of excluded studies and the primary reason for exclusion are provided in Supplement III.

We contacted nine authors by e-mail for additional information. Four authors responded, and one provided additional numerical data [45]. In addition, we received results of an analysis of subgroup effects from an individual patient data (IPD) meta-analysis of five of the included RCTs, in response to an author request [2–6, 46].

### Study characteristics

The eligible RCTs/subgroup analyses of RCTs (in the following all only called RCTs), included 63,374 participants in total. Table 1 shows the characteristics of the included RCTs (for detailed characteristics see supplemental Table IV).

**Table 1:** Characteristics of included studies

Study	Patients (Intervention/Comparison)	Intervention and comparison	Available outcomes
VKA comparison			
ARISTOTLE	**Patient characteristics 65 to < 75y/ ≥ 75y** Age group n(%) 65 to < 75 years 7052(39) ≥ 75 years 5678(31) Female n(%) 2525(35.8)/2396(42.2) Weight [kg] Mean(SD) 84.1(19.2)/76.5(16.4) HAS-BLED-Score n(%) 1 2008(28.5)/1322(23.3) 2 3078(43.6)/2442(43.0) ≥ 3 1966(27.9)/1914(33.7) Renal function by Cockcroft–Gault, n(%) Normal (> 80 ml/min) 2761(39.2)/597(10.5) Mild impairment (> 50 to 80 ml/min) 3511(49.8)/2922(51.5) Moderate impairment (> 30 to 50 ml/min) 713(10.1)/1906(33.6) Severe impairment (≤ 30 ml/min) 40(0.6)/222(3.9)	**Intervention** Apixaban, 5 mg twice daily **Dose reduction** 2.5 mg twice daily for patients with two or more of the following factors: age ≥ 80 years, bodyweight ≤ 60 kg, serum creatinine ≥ 133 μmol/L (≥ 1.5 mg/dL) **Comparison** Dose-adjusted (INR 2–3) warfarin	Mortality Major or clinically relevant bleeding Hospitalisation
ENGAGE AF-TIMI 48	**Patient characteristics age category 65—74y/ ≥ 75y** Age [y] Median(IQR) 70(67.0–72.0)/79(76.0–82.0) Female n(%) 2753(39)/3777(45) Weight[kg] Median(IQR) 83.0(71.0–95.7)/76.0(65.9–86.5) HAS-BLED-Score ≥ 3 n(%) 4129(58)/4779(56) Renal function; CrCl (mL/min) Median(IQR) 74(60–90)/56(45–69)	**Intervention 1** Edoxaban 60 mg once-daily **Intervention 2** Edoxaban 30 mg once-daily **Dose reduction** The dose of edoxaban was reduced by 50% if any of the following factors were present at the time of randomization or during the study: calculated CrCl of ≤ 50 mL/min using the Cockcroft-Gault formula, body weight ≤ 60 kg, or the concomitant use of potent P-glycoprotein inhibitors **Comparison** Dose-adjusted (INR 2–3) warfarin	Major or clinically relevant bleeding
ENVISAGE-TAVI AF	**Patient characteristics** Age [y] Mean(SD) 82.1(5.4)/82.1(5.5) Female n(%) 347(48.7)/(331(46.4) Weight [kg] Mean(SD) 74.6(17.9)/76.0(17.3) HAS-BLED-Score NR Renal function CrCl by Cockcroft-Gault formula [ml/min] Mean(SD) 57.9(24.0)/58.6(24.3)	**Intervention** Edoxaban 60 mg once daily **Dose reduction** 30 mg once daily for patients with a creatinine clearance (Cockcroft-Gault formula) of 15—50 ml/min, a body weight of 60 kg or less, and the use of certain P-glycoprotein inhibitors **Control** Dose-adjusted (INR 2–3) vitamin K antagonists; (adjusted to 1.6—2.6 for patients ≥ 70 years of age in Japan)	Mortality Major or clinically relevant bleeding Discontinuation due to adverse events
RE-DUAL PCI	**Patient characteristics** Age [y] Mean(SD) 79.3(3.6) Female n(%) 322(31.4) Weight / BMI [kg/m^2^] NR Modified HAS-BLED-Score Mean(SD) 3.0(0.6) Renal function NR	**Intervention 1** Dabigatran etexilate 110 mg twice daily plus either clopidogrel or ticagrelor **Intervention 2** Dabigatran etexilate 150 mg twice daily plus either clopidogrel or ticagrelor (dabigatran dual) **Comparison** Warfarin + aspirin (≤ 100 mg daily, 1 month when a bare-metal stent was used, and for 3 months when a drug-eluting stent was used) + either clopidogrel or ticagrelor (warfarin triple) **Dose reduction** Elderly patients in non-US countries (≥ 80 years) or Japan (≥ 70 years) were randomized to receive dabigatran 110 mg dual or warfarin triple therapy, whereas younger patients in non-US countries (< 80 years) and Japan (< 70 years), and all US patients irrespective of age, were randomized to dabigatran 110 mg dual, dabigatran 150 mg dual, or warfarin triple therapy	Mortality Major or clinically relevant bleeding
RE-LY	Patient characteristics IG1/IG2/CG* Age [y] Mean(SD) 71.4(8.6)/71.5(8.8)/71.6(8.6) Female n(%) 2150(35.7)/2236(36.8)/2213(36.7) Weight [kg] Mean(SD) 82.9(19.9)/82.5(19.4)/82.7(19.7) HAS-BLED-Score NR Renal function NR	**Intervention 1** Dabigatran 110 mg twice daily **Intervention 2** Dabigatran 150 mg twice daily **Dose reduction** NR **Comparison** Dose-adjusted (INR 2–3) warfarin	Mortality Major or clinically relevant bleeding
ROCKET-AF	**Patient characteristics IG/CG** Age [y] Median(IQR) 79(76–82)/79(76–82) Female n(%) 1446(46.4)/1432(46.1) BMI [kg/m^2^] Median(IQR) 27.4(24.7–30.7)/27.2(24.6–30.4) HAS-BLED-Score NR Renal function, CrCl [ml/min] Median(IQR) 55(44–68)/55(44–68)	**Intervention** Fixed-dose rivaroxaban 20 mg daily **Dose reduction** 15 mg daily in patients with a CrCl of 30 to 49 ml/min **Comparison** Dose-adjusted (INR 2–3) warfarin	Major or clinically relevant bleeding Total bleeding
PIONEER AF PCI	Patient characteristics IG/CG* Age [y] Mean(SD) 70.0(9.1)/69.9(8.7) Female n(%) 174(24.5)/188(26.6) BMI [kg/m^2^] Median(IQR) 28.4(25.6–32.1)/29.0(25.8–32.8) HAS-BLED-Score n(%) 0 2(0.3)/0(0.0) 1 43(6.1)/26(3.7) 2 182(25.7)/182(25.8) 3 294(41.5)/308(43.6) 4 157(22.1)/157(22.2) 5 30(4.2)/31(4.4) 6 1(0.1)/2(0.3) Renal function, CrCl [ml/min] Mean(SD) 77.5(31.8)/80.7(30.0)	**Intervention** Rivaroxaban, 2.5 mg twice daily plus background DAPT (low-dose aspirin (75 to 100 mg per day) and clopidogrel, 75 mg once daily (or ticagrelor, 90 mg twice daily or prasugrel,10 mg once daily in ≤ 15% of participants) for a prespecified duration of 1, 6, or 12 months Participants who received the treatment for 1 or 6 months then received rivaroxaban, 15 mg once daily (or 10 mg once daily if they had moderate renal impairment) plus background single antiplatelet therapy with low-dose aspirin (75 to 100 mg per day) for the remainder of the 12-month treatment period **Comparison** Dose adjusted (INR 2.0–3.0) warfarin once daily plus background DAPT with low-dose aspirin (75 to 100 mg per day) and clopidogrel, 75 mg once daily (or ticagrelor, 90 mg twice daily or prasugrel, 10 mg once daily in ≤ 15% of participants) for a prespecified duration of 1, 6, or 12 months Participants who received the treatment for 1 or 6 months then received dose adjusted (INR 2.0–3.0) warfarin once daily plus background single antiplatelet therapy with low-dose aspirin (75 to 100 mg per day) for the remainder of the 12-month treatment period	Mortality Major bleeding
ENTRUST-AF PCI	Patient characteristics IG/CG* Age [y] Median(IQR) 69(63–77)/70(64–77) Female n(%) 194(26)/192(25) Weight [kg] Median(IQR) 80(71–93)/83(72–94) HAS-BLED-Score Median(IQR) 3.0(2.0–3.0)/3.0(2.0–3.0) Renal function, CrCl [mL/min], Median(IQR) 71.8(53.7–91.1)/71.7(54.0–90.9)	**Intervention** Edoxaban 60 mg once-daily plus clopidogrel, 75 mg once-daily (or prasugrel,5 mg or 10 mg once daily or ticagrelor, 90 mg twice daily) **Dose reduction** Edoxaban 30 mg once daily (for patients with any of the following characteristics: moderate or severe renal impairment (calculated CrCl 15–50 mL/min), bodyweight ≤ 60 kg, or concurrent use of specific potent P-glycoprotein inhibitors (cyclosporine, dronedarone, erythromycin, or ketoconazole) **Comparison** Dose-adjusted (INR 2.0–3.0) VKA plus clopidogrel, 75 mg once daily (or prasugrel, 5 mg or 10 mg once daily or ticagrelor, 90 mg twice daily) for 12 months and aspirin (100 mg once daily) for a minimum of 1 month and up to 12 months	Mortality Major or clinically relevant bleeding
Valkyrie study	**Only patients with end-stage renal disease** **Patient characteristics IG/CG** Age [y] Median(IQR) 79.9(74.4–83.9)/80.3(71.5–84.3) Female n(%) 11(23.9)/19(43.2) BMI [kg/m^2^] Median (IQR) 24.7(22.0–27.5)/25.6(22.3–30.4) HAS-BLED-Score Mean(SD) 4.6(0.8)/4.7(0.9) Renal function All patients had end-stage renal disease treated with chronic hemodialysis thrice weekly	**Intervention** Rivaroxaban 10 mg daily **Dose reduction** NR **Comparison** Adjusted dose (INR 2–3) VKA	Mortality Major or clinically relevant bleeding
Aspirin and placebo comparison			
AVERROES	**Patient characteristics** Age [y] Mean(SD) 80.4(4.3)/80.4(4.2) Female n(%) 433(47.8)/486(50.0) BMI [kg/m^2^] Mean(SD) 26.9(4.9)/27.0(4.7) HAS-BLED-Score NR Renal function, eGFI Mean(SD) 59.6(16.1)/60.6(16.1)	**Intervention** Apixaban 5 mg twice daily **Dose reduction** 2.5 mg twice daily for patients with two or more of the following factors: age ≥ 80 years, body weight ≤ 60 kg, or serum creatinine ≥ 1.5 mg/dl or 133 μmol/l **Comparison** Aspirin 81—324 mg daily	Mortality Major or clinically relevant bleeding Hospitalisation
ELDERCARE-AF	**Patient characteristics IG/CG** Age [y] Mean(SD) 86.7(4.2)/86.4(4.3) Female n(%) 280(56.9)/285(57.9) BMI [kg/m^2^] Mean(SD) 22.1(3.6)/22.2(3.8) HAS-BLED score Mean(SD) 2.3(0.9)/2.4(0.9) Renal function: CrCl [ml/min] Mean(SD) 36.3(14.3)/36.2(14.5)	**Intervention** Edoxaban, 15 mg, once daily **Dose reduction** NR **Comparison** Placebo	Mortality Major or clinically relevant bleeding Minor bleeding Discontinuation due to adverse events

*CG* comparison group; *CrCl* Creatinine Clearance; *DAPT* dual antiplatelet therapy; *HAS-BLED Score* HAS-BLED score reflects the risk of major or clinically relevant bleeding in patients with atrial fibrillation who are receiving anticoagulant therapy, with values ranging from 0 to 9 and with higher scores indicating greater risk; *INR* international normalized ratio; *IG* intervention group; *IQR* interquartile range; *n* number; *NR* not reported; *SD* standard deviation; *VKA* vitamin K antagonist; *Y* years

*includes patients aging < 65 years because demographics were not reported for the elderly subgroup separately

**Clinically significant bleeding: major, minor or bleeds requiring medical attention

Six RCTs (ARISTOTLE [2, 47], AVERROES [3, 48], ELDERCARE [43], ENGAGE [4, 49, 50], RE-LY [5, 51], and ROCKET [6, 52]) included any patient with AF and three trials (RE-DUAL [7, 53], PIONEER [8, 9], and ENTRUST [42]) were conducted in AF-PCI patients [54]. One RCT was conducted in participants with nonvalvular AF on haemodialysis due to end-stage renal disease (Valkyrie [45]) and one RCT (ENVISAGE) in AF patients after transcatheter aortic-valve replacement (TAVR) [44].

The median or mean age was 70 years or older. All RCTs included more men than women. In all RCTs, a significant proportion of the study population had an increased risk of bleeding and suffered from reduced renal function. Average BMI/weight was above the normal in most studies but does not reach severe obesity in any RCT.

Two RCTs compared apixaban with either warfarin or aspirin [2, 3], four edoxaban with either placebo, aspirin, or VKA [4, 42–44], two dabigatran with warfarin [5, 7] and three rivaroxaban with warfarin [6, 8, 45]. In the PCI trials, the patients were treated with antiplatelets in addition to oral anticoagulants [7, 8, 42].

All trials were funded by the pharmaceutical industry.

### Risk of bias of included RCTs

**Fig. 2:**
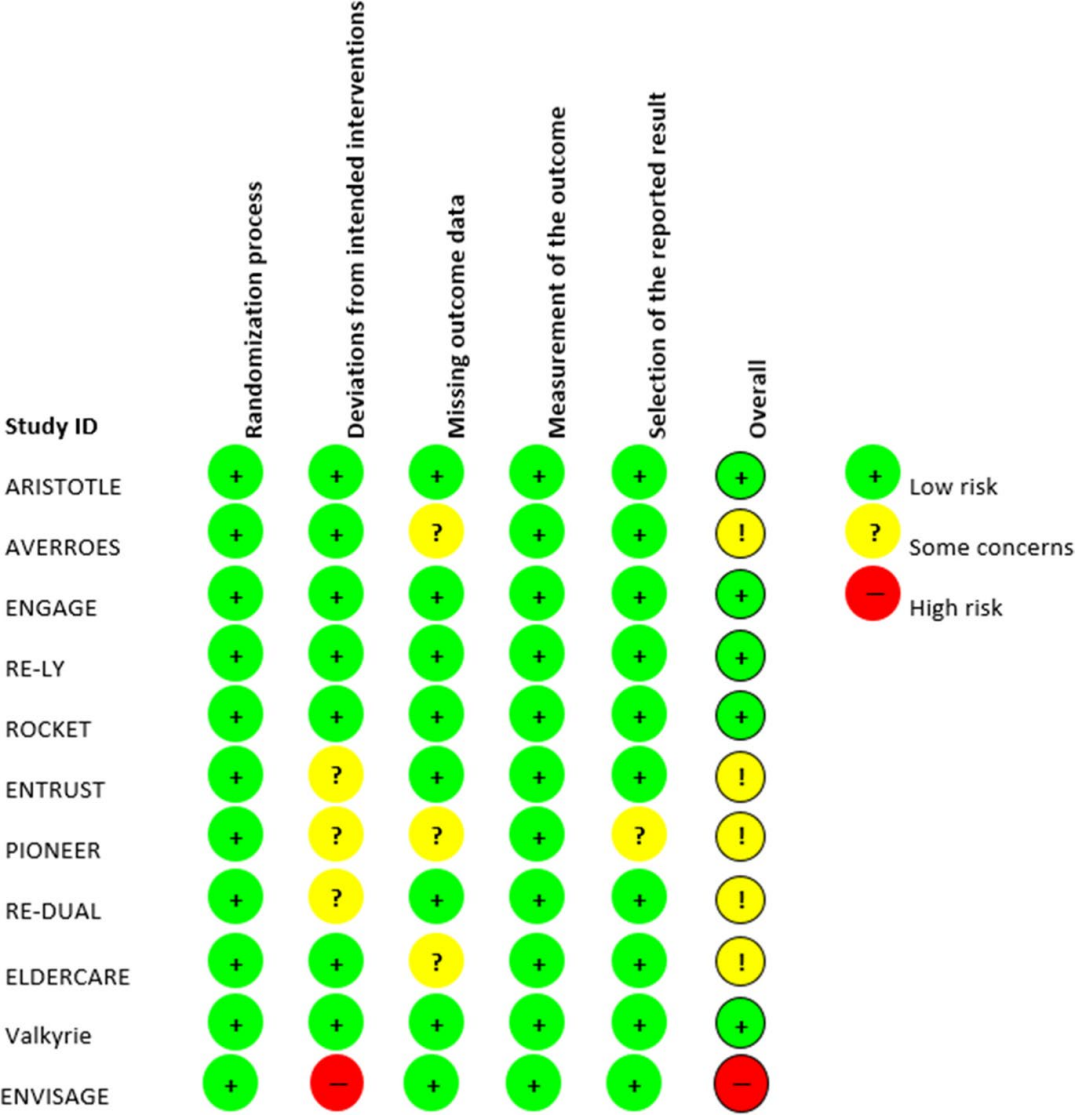
Risk of bias

Figure 2 contains the risk of bias assessment for each individual RCT. Results are presented on study level (not outcome level) because in none of the RCTs the risk of bias differed for different outcomes (e.g., bleeding and falls). For five RCTs we assessed the overall risk of bias to be low [45, 47, 49, 51, 52] and for five RCTs we had some concerns regarding the overall risk of bias [9, 42, 43, 48, 53]. One study was rated to be at high risk of bias [44].

### Bias due to missing evidence

We could not prepare funnel-plots because none of the meta-analyses included at least ten studies. Three publications focused on the elderly but did not report mortality or any adverse event, although this could be expected [49, 52, 53].

### Effects of DOACs on the elderly

**Fig. 3:**
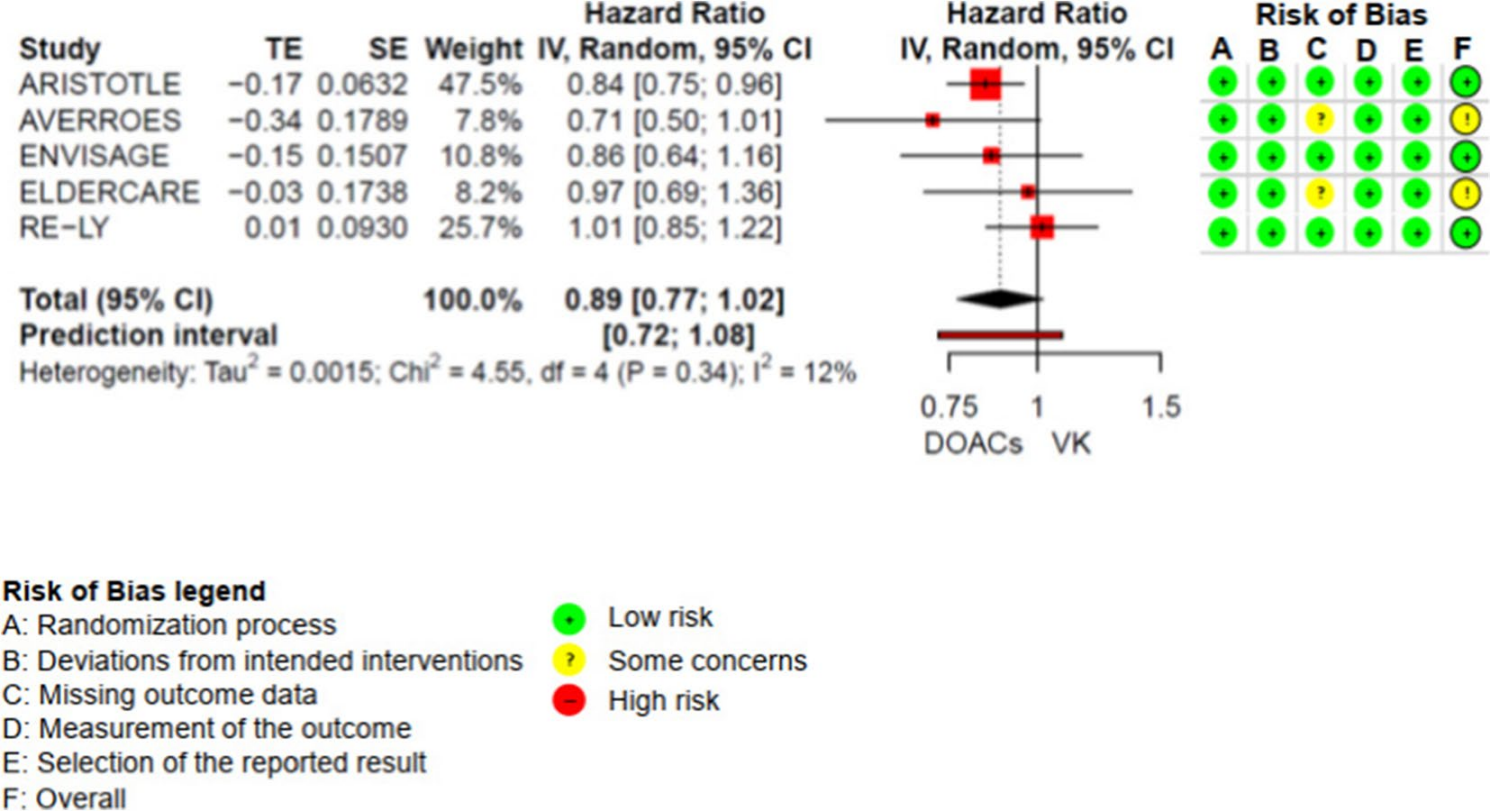
Mortality AF (*n* = 20.904)

**Fig. 4:**
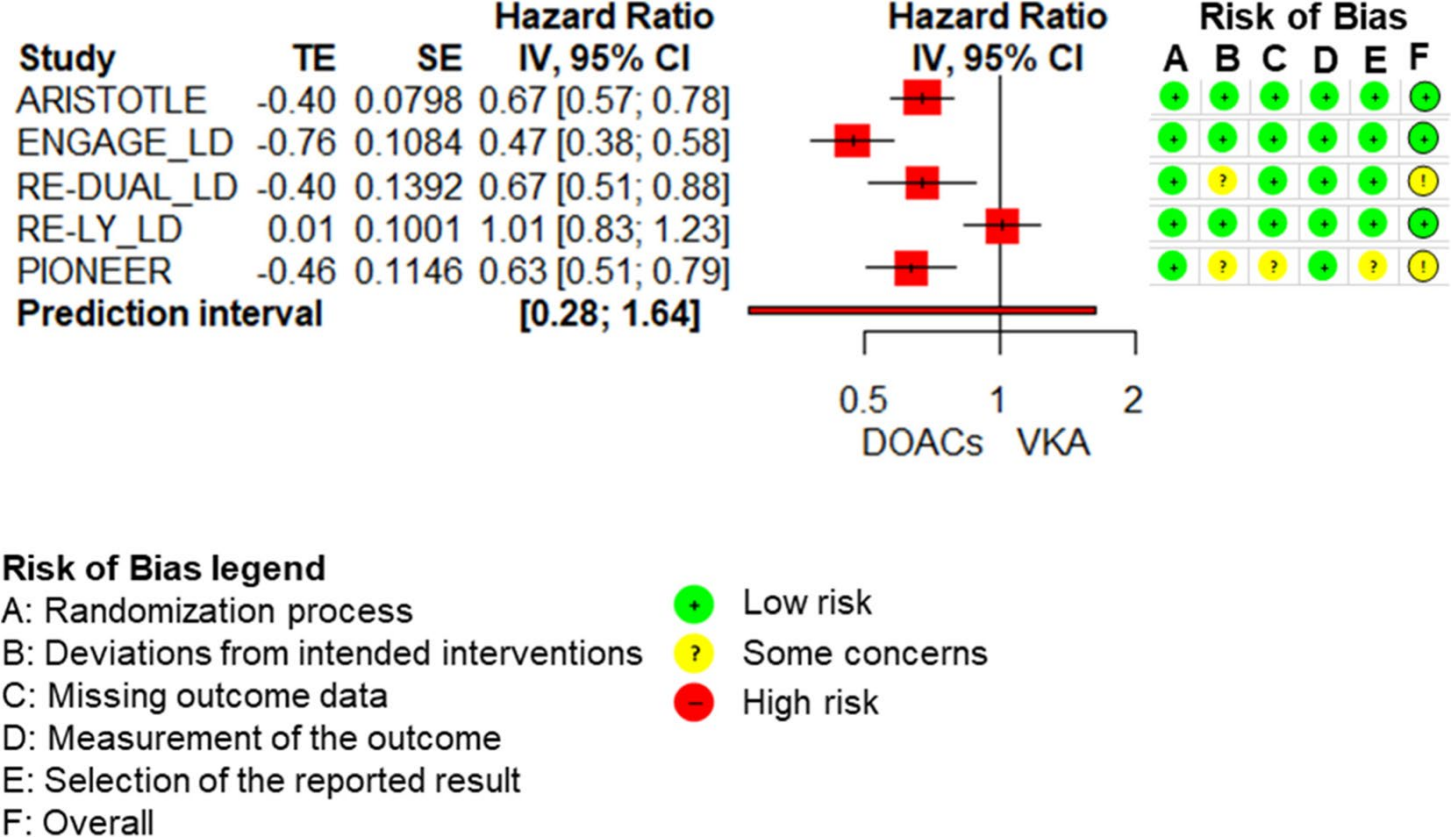
Major bleeding low dose (*n* = 24.997)

The results of the meta-analyses and of each individual RCT included in the meta-analyses are shown in the forest-plots (Fig. 3 Mortality AF (n = 20.904), Fig. 4 Major bleeding low dose (n = 24.997) and supplemental Fig. I). Results of the syntheses with certainty of evidence ratings are presented in the Summary of Findings (Table 2). The RCTs that were not included in the meta-analyses, because they did not match any pre-specified comparison, or because of clinical heterogeneity are presented in supplement V.

**Table 2:**
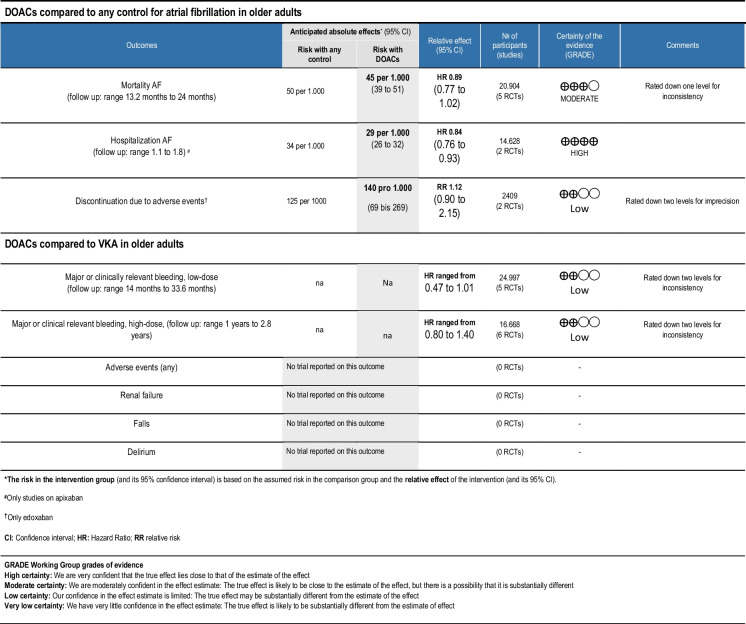
Summary of findings

*The risk in the intervention group (and its 95% confidence interval) is based on the assumed risk in the comparison group and the **relative effect** of the intervention (and its 95% CI)

^**#**^Only studies on apixaban

^**†**^Only edoxaban

*CI* Confidence interval; *HR* Hazard Ratio; *RR* relative risk

**GRADE Working Group grades of evidence**

**High certainty:** We are very confident that the true effect lies close to that of the estimate of the effect

**Moderate certainty:** We are moderately confident in the effect estimate: The true effect is likely to be close to the estimate of the effect, but there is a possibility that it is substantially different

**Low certainty:** Our confidence in the effect estimate is limited: The true effect may be substantially different from the estimate of the effect

**Very low certainty:** We have very little confidence in the effect estimate: The true effect is likely to be substantially different from the estimate of effect

#### Mortality

DOACs probably reduce mortality in elderly patients with AF-only (HR 0.89 95%CI 0.77 to 1.02) [7, 43, 44, 47, 48, 51]. Likewise, in the Valkyrie trial, end stage-renal disease patients with AF receiving low-dose rivaroxaban had numerically lower mortality compared to patients receiving VKAs (RR 0.82 95%CI 0.46 to 1.45) [45]. We could not find any RCT that reported mortality in elderly AF-PCI patients.

#### Major or clinically relevant bleeding

In the meta-analyses, there was statistically significant heterogeneity and therefore the results were not pooled across all included RCTs [9, 42, 44, 47, 49, 51–53]. This was true for both, the meta-analysis on low-dose and on high-dose DOACs. To explore this heterogeneity, we performed post-hoc subgroup analyses. We decided to stratify the analyses according to dose and agent because previous systematic reviews and large real-world studies had suggested that dabigatran and rivaroxaban tend to have a higher bleeding risk than apixaban and edoxaban [13, 15, 16, 18, 20, 55].

For low doses, the separate analyses according to agents did not resolve heterogeneity [9, 47, 49, 51, 53]. A common quantitative measure would therefore be misleading and consequently no meta-analysis was performed, and we only compiled a narrative synthesis. According to this, low-dose DOACs likely reduce bleeding compared to VKAs (HR ranged from 0.47 to 1.01). Likewise, in end-stage renal disease patients, low-dose rivaroxaban decreased major bleeding risk numerically compared to VKAs (RR 0.58 95%CI 0.25 to 1.34) [45]. In the ELDERCARE trial low-dose edoxaban increased major bleeding numerically compared to placebo (HR 1.87 95%CI 0.90 to 3.89) [43]. In the AVERROES trial, apixaban increased major bleeding risk numerically compared to aspirin, but 95%CIs overlapped appreciable benefit and harm (1.21 95%CI 0.69 to 2.12) [48].

For high-dose DOACs, the distinct meta-analyses according to agent did not resolve heterogeneity, and we only performed a narrative synthesize. The risk of major bleeding varied widely (HR ranged from 0.80 to 1.40) [42, 44, 49, 51–53].

#### Subgroup considerations

Table 3 shows the results of the within study subgroup analyses for age. The subgroup analyses indicate that the positive effect on mortality in favour of DOACs might decrease with age. The MCRB risk appears to increase with age, whereby the effect direction in favour of DOACs might reverse in very old people (about 85–90 years). Noticeable, in our meta-analyses of high-dose DOACs the bleeding risk tended to be higher in studies that included older or more morbid patients [42, 44, 49, 51–53]. This was even true for studies on the same agent.

**Table 3:** Subgroup analyses

Mortality		
Study	Hazard ratio (95%CI)	*p*-value of interaction test
ARISTOTLE	Age < 65: 1.07 (0.84 to 1.35) Age 65–75: 0.77 (0.64 to 0.94) Age ≥ 75: 0.91 (0.77 to 1.07)	0.43
AVERROES	Age < 75: 0.99 (0.68 to 1.44) Age ≥ 75: 0.71 (0.50 to 0.99)	0.20
	Age < 85: 0.79 (0.60 to 1.04) Age ≥ 85: 0.80 (0.44 to 1.45)	0.88
RE-LY	**High dose** Age < 75: 0.77 (0.64 to 0.93) Age 75–79: 0.82 (0.63 to 1.07) Age 80–84: 1.16 (0.87 to 1.55) Age ≥ 85: 1.15 (0.74 to 1.79)	0.014
	**Low dose** Age < 75: 0.82 (0.68 to 0.98) Age 75–79: 0.86 (0.66 to 1.11) Age 80–84: 1.09 (0.80 to 1.47) Age ≥ 85: 1.37 (0.89 to 2.11)	0.026
IPD (RE-LY, ROCKET, AVERROES, ARISTOTLE, ENGAGE)	HR (smaller favours DOACS) decrease with age	0.079
Major bleeding		
Study	Hazard ratio (95%CI)	p-value of interaction test
ARISTOTLE	Age < 65:0.78 (0.55 to 1.11) Age 65–75: 0.71 (0.56 to 0.89) Age ≥ 75: 0.64 (0.52 to 0.79)	0.63
AVERROES	Age < 75: 1.14 (0.58 to 2.30) Age ≥ 75: 1.21 (0.69–2.12)	0.90
	Age < 85: 1.21 (0.74 to 1.99) Age ≥ 85: 0.96 (0.38 to 2.39)	0.65
ELDERCARE	Age ≤ 85: 5.31 (0.62 to 45.21) Age ≥ 86: 1.49 (0.67 to 3.29)	NR
ENGAGE	**High dose** Age < 65: 0.81 (0.58 to 1.12) Age 65–74: 0.75 (0.60 to 0.94) Age ≥ 75: 0.83 (0.70 to 0.99)	0.78
	**Low dose** Age < 65: 0.40 (0.27 to 0.61) Age 65–74: 0.48 (0.37 to 0.62) Age ≥ 75: 0.47 (0.38 to 0.58)	0.75
RE-LY	**High dose** Age < 75: 0.70 (0.57 to 0.86) Age 75–79: 1.04 (0.81 to 1.35) Age 80–84: 1.41 (1.02 to 1.94) Age ≥ 85: 1.22 (0.74 to 2.02)	< 0.001
	**Low dose** Age < 75: 0.62 (0.50 to 0.77) Age 75–79: 0.93 (0.71 to 1.21) Age 80–84: 1.18 (0.84 to 1.65) Age ≥ 85: 1.01 (0.59 to 1.73)	< 0.001
ROCKET-AF	Age < 75: 0.96 (0.78 to 1.19) Age ≥ 75: 1.11 (0.92 to 1.34)	0.336
IPD (RE-LY, ROCKET, AVERROES, ARISTOTLE, ENGAGE)	HR (smaller favours DOACS) increases with age. HR < 1 (favours) DOACs until 90 years and reverses (> 1 favours VKAs) thereafter	0.031
ENVISAGE	RR (95% CI) Age < 85: 1.16 (0.91 to 1.48) Age ≥ 85: 0.91 (0.65 to 1.27)	NR
Major OR clinically relevant non-major bleeding		
Study	Hazard ratio (95%CI)	Interaction p-value
ENTRUST	HR (smaller favours DOACs) slightly increases with age	0.914
RE-DUAL	**High dose** Age < 75: 0.57 (0.44 to 0.74) Age ≥ 75: 1.21 (0.83 to 1.77)	0.001
	**Low dose** Age < 75: 0.67 (0.51 to 0.89) Age ≥ 75: 0.40 (0.30 to 0.54)	0.012
Clinically significant bleeding		
Study	Hazard ratio (95%CI)	Interaction p-value
PINOEER	**High Dose** Age < 75: 0.56 (0.41 to 0.77) Age ≥ 75: 0.62 (0.42 to 0.90)	0.712
	**High Dose** Age < 65: 0.57 (0.34 to 0.98) Age ≥ 65: 0.60 (0.46 to 0.78)	0.889
	**Low Dose** Age < 75: 0.61 (0.45 to 0.82) Age ≥ 75: 0.66 (0.45 to 0.95)	0.753
	**Low Dose** Age < 65: 0.70 (0.43 to 1.16) Age ≥ 65: 0.62 (0.47 to 0.81)	0.678

Subgroup analyses for major bleeding according to all AF patients versus AF-PCI patients [8, 42, 53] do not change the results (data not shown). An explorative analysis of bleeding risk according to body part suggested that DOACs increase the risk of gastrointestinal bleeding but reduce the risk of intracranial bleeding numerically (data not shown).

#### Secondary outcomes

Apixaban likely reduces overall hospitalisations (HR 0.84 95%CI 0.76 to 0.93) [47, 48]. In the ELDERCARE trial the difference in hospitalizations was negligible (RR 1.02 95%CI 0.67 to 1.58) [43]. Discontinuations due to adverse events, were numerically slightly increased in patients taking VKAs compared to edoxaban but the effect is uncertain because of statistical imprecision (RR 1.12 95%CI 0.58 to 2.15) [43, 44].

We did not find any RCT that reported on hospitalisations in AF-PCI patients. There is no evidence from RCTs on overall adverse events, renal failure, falls or delirium in elderly patients with AF treated with DOACs.

#### Sensitivity analyses

We performed no sensitivity analysis according to risk of bias because none of the RCTs was assessed to be at risk of bias in the randomisation domain.

Sensitivity analyses of meta-analyses including < 4 RCTs and few numbers of events were not possible because the beta-binomial model is a one-stage model, which requires data that allow to reconstruct a contingency table, but for almost all RCTs only aggregated data (e.g., HRs) were available.

## Discussion

### Summary and interpretation in consideration of other evidence

Our systematic review on safety outcomes shows that DOACs probably reduce mortality in elderly AF-only patients to a larger extent than VKAs. The findings were consistent across different agents and different doses agree with previous results of RCTs on all age groups, which suggests that the global effectiveness of DOACs in AF is not significantly influenced by age and a positive benefit-risk ratio of DOACs in comparison to VKAs does also exist in the older population with AF-only [13, 25, 46]. Likewise, the effects seem consistent across different DOACs [56]. In the population with AF, the lower risk for bleeding in the low-dose treatment groups is apparently not counterbalanced by a higher risk for lethal thromboembolic events. In the high-dose treatment groups a significantly higher bleeding risk seems to exist for dabigatran and rivaroxaban, which might be explained by the different extent of renal elimination. The major bleeding risk for edoxaban was not consistent. In the ENGAGE and ENTRUST trials the bleeding risk using DOACs was reduced compared to VKAs. In contrast, in the ENVISAGE study the risk was increased [42, 44]. This finding might be explained by the higher average age and higher cardiovascular morbidity compared to both other studies.

We found no RCT that reported on mortality for AF-PCI patients, however meta-analyses in the entire population, i.e. not only elderly, showed that mortality in the DOACs group was not statistically significantly higher than in the VKA group [42, 57]. Apparently, in this population, the approach of combining DOACs with only one antiplatelet agent (instead of dual antiplatelet therapy = DAPT) in comparison to the efficacy and safety with VKA plus DAPT results in a lower risk for bleeding, but a higher risk for thromboembolic or coronary events.

Studies based on real-world data showed heterogeneous results for mortality when using DOACs compared to VKAs in AF patients [14–17]. The studies neither distinguished between AF-only and AF-PCI patients nor patients with different heart disease severity in general. Remarkably, in these observational studies, morbidity due to cardiovascular diseases was high and more similar to the PCI population than to the AF-only population in our review. The differences in morbidity, in particular the probable differences in proportion of PCI-patients, might be one explanation for the heterogeneous results for the effectiveness of DOACs in studies based on real-world data and also for the tendency of a weaker impact of DOACs on mortality under routine conditions compared to the RCTs on AF-only patients [14, 15, 57].

Another explanation for the heterogeneous findings could be the type of VKA used. In large real-world studies performed in the USA and Denmark, taking DOACs was associated with fewer deaths compared to VKAs [16, 17]. In contrast, similar studies performed in Germany showed higher mortality compared to VKAs [15]. The reason for this difference could be the different VKA prescribing practices; in the USA, warfarin is mainly, whereas phenprocoumon is prescribed in Germany. Pharmacological studies showed that for long-term use, phenprocoumon is preferable compared to warfarin because phenprocoumon patients more often have an INR in the therapeutic range [58]. Conspicuously, the patterns of mortality and major bleeding risk appear to agree, concrete studies showing higher major bleeding risk tend to show less favourable results for mortality, indicating that at least a part of the differences in mortality might be explained by death as a result of major bleeding. However, to our knowledge no RCTs exist that directly compare phenprocoumon to warfarin.

We found that low-dose DOACs probably decrease MCRB compared to VKA in AF-only and similarly in AF-PCI patients but could not quantify this reduction reliably because of statistical heterogeneity. The heterogeneity could neither be fully explained by subgroup analyses of patient type (AF-only vs. AF-PCI) nor by subgroup analysis on drug type. All but one RCT showed reduced MCRB and in one RCT the bleeding rates were comparable between DOACs (dabigatran) and warfarin. In the two RCTs on low-dose dabigatran, the PCI trial showed lower major bleeding risk using dabigatran compared to warfarin, which is probably explained by the concomitant therapy with only one antiplatelet agent in the DOACs group and DAPT in the warfarin group. Consistent with real-world studies, edoxaban and even more apixaban showed the lowest risk for MCRB while heterogeneity is large [15, 16, 56].

Preliminary evidence, showed numerically more MCRB when taking low-dose DOACs compared to aspirin and placebo in AF-only patients [43, 48]. Furthermore, the RCT of low-dose rivaroxaban in AF-only patients with end-stage renal disease provides a hint that the results for MCRB in this population might be similar to the results in the elderly in general, meaning that the current evidence from RCTs does not indicate that in patients with end-stage renal disease DOACs should not be prescribed in general [45].

For high-dose DOACs the MCRB risk seems to depend on the agent. This seems to be true both for AF-only and AF-PCI patients. Rivaroxaban and dabigatran increased the bleeding risk. Again, the real-world studies found heterogeneous results for these drugs [14–18]. However, it must be considered that the impact of different doses of DOACs was not analysed in these observational studies. Considering that the quality of evidence for our findings is high and considering results of previous analyses on the influence of dosing, it appears plausible that the different doses are an additional important explanation for the heterogeneous findings on safety of DOACs in the real-world [13]. For high-dose edoxaban, MCRB risk was very heterogeneous. One explanation could be the higher age and morbidity in the ENVISAGE trial, compared to all other trials [44].

### Applicability of findings

Comparing our study population to the patient population from real-world studies confirmes that our population mirrors the patients in routine care quite well. Therefore, none of the RCTs was down-graded due to limited applicability in the certainty of evidence assessment. Notwithstanding, the AF-only patients still tend to be less morbid and comorbid than patients in real-world studies [14–18, 59]. Moreover, all but two studies that compared DOACs to VKAs used warfarin whereas in some countries other VKAs are mainly prescribed, which limits applicability of the results to these countries [60].

### Quality of the evidence

The risk of bias of the body of evidence was low. The main limitation of the certainty of evidence for mortality was statistical imprecision. In each individual study, DOACs were at least as effective as VKAs in preventing mortality but effect sizes apparently varied. Although the difference in effectiveness between studies might be simply due to random error, we could not exclude that this is due to the different DOACs. In addition, for low-dose DOACs, the certainty of evidence on MCRB is limited by unexplained heterogeneity.

The evidence in this systematic review is incomplete regarding several safety outcomes including overall adverse events, adverse events leading to discontinuation, and adverse events particularly relevant for the elderly such as delirium or falls.

### Limitations

One limitation of this systematic review is the literature search. We decided to identify the evidence using previous systematic reviews to speed up the review process. We anticipated that this is a reasonable shortcut considering the very huge number of systematic reviews on DOACs and therefore low risk of missing relevant literature when relying on previous systematic literature searches. In addition, some might argue that the findings are limited because a large part of them stems from subgroup analyses from RCTs on elderly. However, most of the RCTs were very large and additionally stratified the randomisation for age and adjusted the analyses for important prognostic factors. Therefore, it seems improbable that this approach has introduced bias.

## Conclusion

### Implications for research

There is an important research gap on overall adverse events and particularly outcomes that are relevant for older adults such as falls, fractures or renal impairment in AF-patients in general [61]. In addition, for AF-PCI patients high quality data on mortality is lacking. Studies on these outcomes are necessary for sufficient balancing of the benefits and harms of DOACs use in elderly patients, especially given the low absolute mortality and MCRB risk. Moreover, patient characteristics which might explain the heterogeneity in the real-world, such as very high age, body weight, renal function, severe and multi-morbidity, should be further explored because better information on these potential predictors could contribute to an improved individualization of anticoagulation therapy.

### Implications for practice

No conclusive judgement on the safety of DOACs in older adults is possible because of the lack of RCTs assessing overall adverse events and outcomes relevant in the elderly (e.g., fractures, delirium) [61]. Our data and external evidence from real-world studies suggest that the bleeding risk depends on agent, dose and age. Moreover, the impact of DOACs on mortality and hospitalization probably depends on patient type (AF-only vs. AF-PCI). Similarly to previous systematic reviews on all age groups, we found that low-dose DOACs probably decrease mortality in older AF-only patients. Moreover, apixaban and low-dose edoxaban are associated with fewer MCRB events compared to VKAs [13]. For dabigatran and rivaroxaban, the risk of MCRB varies depending on dose. Moreover, subgroup analyses indicate that in the very old (≥ 85 years) the bleeding risk of DOACs in general, but especially for dabigatran and rivaroxaban might be even higher than for VKAs. The uncertainty due to heterogeneous bleeding risk and the limited impact of DOACs on absolute mortality, suggest once again that the individual anticoagulation treatment choice should cautiously balance the individual patient’s benefit-risk profile, especially in the very old or morbid patient.

### Supplementary Information

Below is the link to the electronic supplementary material.

Supplementary file1 (DOCX 96 KB)

## Data Availability

The datasets used and/or analysed during the current study are available from the corresponding author on reasonable request.

## Abbreviations

*AF*: Atrial fibrillation
*DOAC*: Direct oral anticoagulants
*MCRB*: Major or clinically relevant bleeding
*PCI*: Percutaneous coronary intervention
*RCT*: Randomised controlled trial
*TAVR*: Transcatheter aortic-valve replacement
*VKA*: Vitamin K antagonists
