# Supplementary material for: Safety outcomes of direct oral anticoagulants in older adults with atrial fibrillation: a systematic review and meta-analysis of (subgroup analyses from) randomized controlled trials
Source: GeroScience. 2023 Jun 1;46(1):923–44. doi: 10.1007/s11357-023-00825-2 (PMC10828375; doi:10.1007/s11357-023-00825-2)

**Safety of direct oral anticoagulants in older adults with atrial fibrillation: a systematic review and meta-analysis of (subgroup analyses from) randomized controlled trials**

Katharina Doni, Stefanie Bühn, Alina Weise, Nina-Kristin Mann, Simone Hess, Susanna Salem, Andreas Sönnichsen, Dawid Pieper, Petra Thürmann, Tim Mathes*

* Corresponding author

Affiliation of corresponding author:

Institute for Research in Operative Medicine, School of Medicine, Faculty of Health, Witten/Herdecke University, Witten, Germany

Department of Clinical Pharmacology, School of Medicine, Faculty of Health, Witten/Herdecke University, Witten, Germany

e-mail: [Tim.Mathes@uni-wh.de](mailto:Tim.Mathes@uni-wh.de)

**Supplement**

**Supplement I: Embase search strategy**

| ('aged'/de OR 'frail elderly'/de OR 'geriatrics'/de OR 'very elderly'/de OR 'aging'/de OR (geriatric* OR elder* OR old* OR ageing OR aging):ti,ab)  AND ('atrial fibrillation'/exp OR “atrial fibrillation”/de OR 'thromboembolism'/exp OR thromboembolism/de OR (“atrial fibrillation” OR “atrium fibrillation” OR “auricular fibrillation” OR thromboembolism):ti,ab OR (thromboembolic NEXT/1 (complication OR disease OR process)):ti,ab OR (thromboembolus OR thromboemboly):ti,ab)  AND ('anticoagulant agent'/de OR “oral anticoagulants”/de OR (anticoagulant* OR “anti-coagulant*”):ti,ab OR “vitamin k antagonist*”/de OR “antivitamin k”/de OR “anti vitamin k”:ti,ab OR edoxaban/de OR (edoxaban OR lixiana):ti,ab OR warfarin/de OR (warfarin OR coumadin):ti,ab OR phenprocoumon/de OR phenprocoumon*:ti,ab OR acenocoumarol/de OR (acenoc$umarol* OR acenoc$umarin OR nic$umalon* OR nitro?arfari$n OR sintrom):ti,ab OR 'antithrombin'/exp OR antithrombin/de OR antithrombin*:ti,ab OR “new anticoagulants”/de OR “new anticoagulant*”:ti,ab OR 'thrombin inhibitor'/de OR “direct thrombin inhibitors”/de OR “direct thrombin inhibitor*”:ti,ab OR “dabigatran etexilate”/de OR (“dabigatran etexilate” OR pradax* OR rendix):ti,ab OR dabigatran/de OR dabigatran:ti,ab OR 'blood clotting factor 10a inhibitor'/de OR “direct factor xa inhibitors”/de OR “direct factor xa inhibitor*”:ti,ab OR rivaroxaban/de OR (rivaroxaban OR xarelto):ti,ab OR apixaban/de OR (apixaban* OR eliquis):ti,ab)  AND ((((adverse OR dangerous OR harmful OR side OR undesirable) NEXT/1 (complication* OR consequence* OR effect* OR event* OR reaction*)):ti,ab) OR 'inappropriate medication':ti,ab OR 'inappropriate medications':ti,ab OR 'inappropriate drug':ti,ab OR 'inappropriate drugs':ti,ab OR 'inappropriate prescription':ti,ab OR 'inappropriate prescribing':ti,ab OR 'potentially inappropriate medication'/de OR 'adverse drug reaction'/de OR 'quality of life'/de OR 'patient satisfaction'/de OR 'satisfaction'/de OR 'life satisfaction'/de OR 'patient preference'/de OR 'daily life activity'/de OR 'quality adjusted life year'/de OR 'personal autonomy'/de OR 'happiness'/de OR 'self-concept'/de OR 'social support'/de OR 'family relation'/de OR 'religion'/de OR 'quality of life':ti,ab OR qol:ti,ab OR 'personal autonomy':ti,ab OR 'happiness':ti,ab OR 'life quality':ti,ab OR 'quality adjusted life year*':ti,ab OR 'patient satisfaction':ti,ab OR 'patient preference*':ti,ab OR 'activities of daily living':ti,ab OR 'adl disability'/de OR 'mortality'/de OR death:ti,ab OR died:ti,ab OR mortality:ti,ab OR bleed*:ti,ab OR 'hospitalization'/de OR hospitali?ation:ti,ab OR 'functional status'/de OR headache:ti,ab OR diarrhea:ti,ab OR constipation:ti,ab OR 'cognitive impairment':ti,ab OR 'cognitive status':ti,ab OR 'functional status':ti,ab OR 'functional impairment':ti,ab OR 'life expectancy'/de OR 'drug toxicity'/de OR 'drug toxicity and intoxication'/de OR 'safety'/de OR 'patient safety'/de OR ((drug NEXT/1 (toxicity OR intoxication)):ti,ab) OR “patient safety”:ti,ab OR 'adverse event'/de OR 'kidney failure'/de OR (((kidney OR renal) NEXT/1 (failure OR insufficiency)):ti,ab) OR 'cardiovascular event'/exp OR “cardiovascular event”:ti,ab OR 'heart infarction'/de OR (“heart attack” OR “heart infarction” OR “myocardial infarction” OR “cardiac infarct” OR “cardiac infarction” OR “cardial infarct” OR “heart infarct” OR “myocardial infarct” OR “myocardium infarct” OR “myocardium infarction”):ti,ab OR (stroke OR apoplex* OR “cerebrovascular accident” OR cva OR “brain attack”):ti,ab OR 'transient ischemic attack'/de OR “transient isch$emic attack”:ti,ab OR 'falling'/de OR fall*:ti,ab OR 'delirium'/de OR (delirium OR delier OR delire OR deliria OR “deliri$us state” OR “deliri$us syndrome” OR “delirium acutum”):ti,ab)  AND (random*:ab,ti OR placebo*:de,ab,ti OR (double NEXT/1 blind*):ab,ti)  AND (english:la OR german:la)  AND ('article'/it OR 'article in press'/it OR 'review'/it)  AND ([1-10-2015]/sd)  NOT ('case report'/de OR 'in vitro study'/de OR 'animal experiment'/de) |
| --- |

**Supplement II: data items extracted**

1. bibliometric information:

1.1. name of first author

1.2. year of publication

2. trial-specific information:

2.1. trial name

2.2. source of funding

2.3. number and geographic regions of recruiting centers

2.4. NCT number, i.e., unique identification code given to each clinical study record registered on ClinicalTrials.gov

2.5. enrollment start and end dates

2.6. length of treatment period

2.7. length of participant follow-up

3. participant-specific information:

3.1. number of randomized participants aged 65 years or older

3.2. main inclusion and exclusion criteria

3.3. baseline characteristics of participants such as age, gender, and body-weight/Body Mass Index (BMI)

3.4. relevant clinical characteristics and comorbidities at baseline

3.5. comedication at baseline

4. intervention(s) and comparison intervention(s)

4.1. drugs with:

4.2. doses

4.3. frequency

4.4 dose reductions with criteria

5. outcomes:

5.1. all-cause mortality

5.2. major bleeding as defined by specific trials

5.3. data on bleeding risk according the localisation (gastrointestinal, intracranial) to further explore the effect of DOACs on major bleeding.

5.4. any adverse event

5.5. discontinuation due to adverse events

5.6. renal failure

5.7. delirium

5.8. falls

5.9. length of follow-up (if necessary for each outcome separately)

6. Funding source

**Supplement III: List of excluded studies with reasons**

| **Excluded publications** | **Primary reason for exclusion** |
| --- | --- |
| Agnelli G, Buller HR, Cohen A et al.; AMPLIFY-EXT Investigators. Apixaban for extended treatment of venous thromboembolism. N Engl J Med 2013;368:699–708. | P |
| Agnelli G, Buller HR, Cohen A, Curto M, Gallus AS, Johnson M, Masiukiewicz U, Pak R, Thompson J, Raskob GE, Weitz JI; AMPLIFY Investigators. Oral apixaban for the treatment of acute venous thromboembolism. N Engl J Med. 2013;369:799–808. doi: 10.1056/NEJMoa1302507 | P |
| Agnelli G, Gallus A, Goldhaber SZ, et al. Treatment of proximal deepvein thrombosis with the oral direct factor Xa inhibitor rivaroxaban (BAY 59-7939): the ODIXa-DVT (Oral Direct Factor Xa Inhibitor BAY 59-7939 in Patients With Acute Symptomatic Deep-Vein Thrombosis) study. Circulation 2007;116:180–187 | P |
| Alexander JH, Lopes RD, James S, et al. Apixaban with antiplatelet therapy after acute coronary syndrome. N Engl J Med 2011; 365:699–708. | P |
| APPRAISE Steering Committee and Investigators, Alexander JH, Becker RC, et al. Apixaban, an oral, direct, selective factor Xa inhibitor, in combination with antiplatelet therapy after acute coronary syndrome: results of the Apixaban for Prevention of Acute Ischemic and Safety Events (APPRAISE) trial. Circulation 2009;119:2877–2885. | P |
| Benavente O, Hart R, Koudstaal P, Laupacis A, McBride R. Antiplatelet therapy for preventing stroke in patients with atrial fibrillation and no previous history of stroke or transient ischemic attacks. In: Warlow C, Van Gijn J, Sandercock P, eds. Stroke Module of the Cochrane Database of Systematic Reviews. Oxford, UK: The Cochrane Collaboration; 1999. | I |
| Boston Area Anticoagulation Trial for Atrial Fibrillation Investigators. The eJect of low-dose warfarin on the risk of stroke in nonrheumatic atrial fibrillation. New England Journal of Medicine 1990;323:1505-11. | I |
| Bousser MG, Bouthier J, Bu¨ller HR, et al; Amadeus Investigators. Comparison of idraparinux with vitamin K antagonists for prevention of thromboembolism in patients with atrial fibrillation: a randomised, open-label, non-inferiority trial. Lancet. 2008;371(9609):315-321. | P |
| Bu¨ller HR, Prins MH, Lensin AW, Decousus H, Jacobson BF, Minar E, et al; EINSTEIN–PE Investigators. Oral rivaroxaban for the treatment of symptomatic pulmonary embolism. N Engl J Med. 2012;366:1287-97. [PMID: 22449293] | P |
| Buller H, Deitchman D, Prins M, Segers A. Efficacy and safety of the oral direct factor Xa inhibitor apixaban for symptomatic deep vein thrombosis. The Botticelli DVT dose-ranging study. J Thromb Haemost. 2008;6:1313–1318. | P |
| Buller HR, Decousus H, Grosso MA, Mercuri M, Middeldorp S, Prins MH, Raskob GE, Schellong SM, Schwocho L, Segers A, Shi M, Verhamme P, Wells P. Edoxaban versus warfarin for the treatment of symptomatic venous thromboembolism. N Engl J Med. 2013;369:1406–1415. | P |
| Buller HR, Lensing AW, Prins MH, et al. A dose-ranging study evaluating once-daily oral administration of the factor Xa inhibitor rivaroxaban in the treatment of patients with acute symptomatic deep vein thrombosis: the Einstein-DVT Dose-Ranging Study. Blood 2008;112:2242–2247. | P |
| Chung N, Jeon HK, Lien LM, et al. Safety of edoxaban, an oral factor Xa inhibitor, in Asian patients with non-valvular atrialfibrillation. Thromb Haemost. 2011;105:535–544 | follow-up length ≥ 12 months |
| Cohen AT, Spiro TE, B€uller HR et al.; MAGELLAN Investigators. Rivaroxaban for thromboprophylaxis in acutely ill medical patients. N Engl J Med 2013;368:513–523 | P |
| Committee R-MW, Ginsberg JS, Davidson BL, et al. Oral thrombin inhibitor dabigatran etexilate vs North American enoxaparin regimen for prevention of venous thromboembolism after knee arthroplasty surgery. J Arthroplasty 2009;24:1–9. | P |
| Connolly S, Pogue J, Hart R, et al; ACTIVE Writing Group of the ACTIVE Investigators. Clopidogrel plus aspirin versus oral anticoagulation for atrial fibrillation in the Atrial fibrillation Clopidogrel Trial with Irbesartan for prevention of Vascular Events (ACTIVE W): a randomised controlled trial. Lancet. 2006;367(9526):1903-1912. | P |
| Connolly SJ, Ezekowitz MD, Yusuf S, Reilly PA, Wallentin L; Randomized Evaluation of Long-Term Anticoagulation Therapy Investigators. Newly identified events in the RE-LY trial [Letter]. N Engl J Med. 2010;363:1875-6.[PMID: 21047252] | S |
| Connolly SJ, Laupacis A, Gent M, Roberts RS, Cairns JA, Joyner C, et al. Canadian Atrial Fibrillation Anticoagulation (CAFA) Study. Journal of the American College of Cardiology 1991;18:349-55. | I |
| Connolly SJ, Pogue J, Hart RG, et al. Effect of clopidogrel added to aspirin in patients with atrial fibrillation. N EnglJMed. 2009;360:2066–2078 // The ACTIVE Investigators. Effect of clopidogrel added to aspirin in patients with atrial fibrillation. N Engl J Med 2009;360:2066–78. | O |
| Diener HC, Connolly SJ, Ezekowitz MD, Wallentin L, Reilly PA, et al. (2010) Dabigatran compared with warfarin in patients with atrial fibrillation and previous transient ischaemic attack or stroke: a subgroup analysis of the RE-LY trial. Lancet Neurol 9: 1157–1163. | P |
| Diener HC, Lowenthal A. Antiplatelet therapy to prevent stroke: risk of brain hemorrhage and efficacy in atrial fibrillation. J Neurol Sci. 1997;153:112. | S |
| Easton JD, Lopes RD, Bahit MC, Wojdyla DM, Granger CB, et al. (2012) Apixaban compared with warfarin in patients with atrial fibrillation and previous stroke or transient ischaemic attack: a subgroup analysis of the ARISTOTLE trial. Lancet Neurol 11: 503–511. | P |
| Edvardsson N, Juul-Mo¨ller S, Omblus R, Pehrsson K. Effects of low-dose warfarin and aspirin versus no treatment on stroke in a medium-risk patient population with atrial fibrillation. J Intern Med. 2003;254:95-101. [PMID:12823646] | I |
| EINSTEIN Investigators, Bauersachs R, Berkowitz SD, Brenner B et al. Oral rivaroxaban for symptomatic venous thromboembolism. N Engl J Med 2010;363:2499–2510. | P |
| Eriksson BI, Borris L, Dahl OE, et al. Oral, direct Factor Xa inhibition with BAY 59-7939 for the prevention of venous thromboembolism after total hip replacement. J Thromb Haemost 2006;4:121–128. | P |
| Eriksson BI, Borris LC, Dahl OE, et al. A once-daily, oral, direct Factor Xa inhibitor, rivaroxaban (BAY 59-7939), for thromboprophylaxis after total hip replacement. Circulation 2006;114:2374–2381 | P |
| Eriksson BI, Borris LC, Dahl OE, et al. Dose-escalation study of rivaroxaban (BAY 59-7939)–an oral, direct Factor Xa inhibitor–for the prevention of venous thromboembolism in patients undergoing total hip replacement. Thromb Res 2007;120:685–693. | P |
| Eriksson BI, Borris LC, Friedman RJ, et al. Rivaroxaban versus enoxaparin for thromboprophylaxis after hip arthroplasty. N Engl J Med 2008;358:2765–2775. | P |
| Eriksson BI, Dahl OE, Buller HR, et al. A new oral direct thrombin inhibitor, dabigatran etexilate, compared with enoxaparin for prevention of thromboembolic events following total hip or knee replacement: the BISTRO II randomized trial. J Thromb Haemost 2005;3:103–111. | P |
| Eriksson BI, Dahl OE, Huo MH, et al. Oral dabigatran versus enoxaparn for thromboprophylaxis after primary total hip arthroplasty (RE-NOVATE II*). A randomised, double-blind, non-inferiority trial. Thromb Haemost 2011;105:721–729. | P |
| Eriksson BI, Dahl OE, Rosencher N, et al. Dabigatran etexilate versus enoxaparin for prevention of venous thromboembolism after total hip replacement: a randomised, double-blind, non-inferiority trial. Lancet 2007;370:949–956. | P |
| Eriksson BI, Dahl OE, Rosencher N, et al. Oral dabigatran etexilate vs. subcutaneous enoxaparin for the prevention of venous thromboembolism after total knee replacement: the RE-MODEL randomized trial. J Thromb Haemost 2007;5:2178–2185. | P |
| European Atrial Fibrillation Trial Study Group. Secondary prevention in nonrheumatic atrial fibrillation after transient ischaemic attack or minor stroke. EAFT (European Atrial Fibrillation Trial) Study Group. Lancet 1993;342:1255–62. | I |
| Executive Steering Committee on behalf of the SPORTIF III Investigators. Stroke prevention with the oral direct thrombin inhibitor ximelagatran compared with warfarin in patients with non-valvular atrial fibrillation (SPORTIF III): randomised controlled trial. Lancet. 2003;362:1691-8. [PMID: 14643116] // Olsson SB; Executive Steering Committee of the SPORTIF III Investigators. Stroke prevention with the oral direct thrombin inhibitor ximelagatran compared with warfarin in patients with nonvalvular atrial fibrillation (SPORTIF III): randomised controlled trial. Lancet. 2003;362(9397):1691-1698. | I |
| Ezekowitz MD, Bridgers SL, James KE, Carliner NH, Colling CL, Gornick CC, et al.Warfarin in the prevention of stroke associated with nonrheumatic atrial fibrillation. Veterans Affairs Stroke Prevention in Nonrheumatic Atrial Fibrillation Investigators. N Engl J Med. 1992;327:1406-12. | I |
| Ezekowitz MD, Reilly PA, Nehmiz G, et al. Dabigatran with or without concomitant aspirin compared with warfarin alone in patients with nonvalvular atrial fibrillation (PETRO Study). Am J Cardiol 2007;100:1419e1426. | P |
| FFAACS (Fluindione, Fibrillation Auriculaire, Aspirin et Contraste Spontane) Investigators. Anticoagulant (fluindione)-aspirin combination in patients with high-risk atrial fibrillation. A randomized trial (Fluindione, Fibrillation Auriculaire, Aspirin et Contraste Spontane´; FFAACS). Cerebrovasc Dis. 2001;12:245-52. [PMID: 11641591 | I |
| Fuji T, Fuijita S, Ujihira T, et al. Dabigatran etexilate prevents venous thromboembolism after total knee arthroplasty in Japanese patients with a safety profile comparable to placebo. J Arthroplasty 2010;25:1267–1274. | P |
| Fuji T, Fujita S, Tachibana S, et al. A dose-ranging study evaluating the oral factor Xa inhibitor edoxaban for the prevention of venous thromboembolism in patients undergoing total knee arthroplasty. J Thromb Haemost 2010;8:2458–2468. | P |
| Goldhaber SZ, Leizorovicz A, Kakkar AK, et al. Apixaban versus enoxaparin for thromboprophylaxis in medically ill patients. N Engl J Med 2011;365:2167–2177. | P |
| Gullov AL, Koefoed BG, Petersen P, Pedersen TS, Andersen ED, Godtfredsen J, et al. Fixed minidose warfarin and aspirin alone and in combination vs adjusted-dose warfarin for stroke prevention in atrial fibrillation: Second Copenhagen Atrial Fibrillation, Aspirin, and Anticoagulation Study. Arch Intern Med. 1998;158:1513-21. | P |
| Gullov AL, Koefoed BG, Petersen P. Bleeding during warfarin and aspirin therapy in patients with atrial fibrillation: The AFASAK 2 Study. Arch Intern Med 1999;159:1322e1328 | I |
| Halperin J, Wojdyla D, Piccini JP et al. Efficacy and safety of rivaroxaban compared with warfarin among elderly patients with nonvalvular atrial fibrillation in the ROCKET-AF Trial. Stroke 2012;43:A148. | S |
| Hankey GJ, Patel MR, Stevens SR, Becker RC, Breithardt G, Carolei A, Diener HC, Donnan GA, Halperin JL, Mahaffey KW, Mas JL, Massaro A, Norrving B, Nessel CC, Paolini JF, Roine RO, Singer DE, Wong L, Califf RM, Fox KA, Hacke W; ROCKET AF Steering Committee Investigators. Rivaroxaban compared with warfarin in patients with atrial fibrillation and previous stroke or transient ischaemic attack: a subgroup analysis of ROCKET AF. Lancet Neurol. 2012;11:315–322. | P |
| Harenberg J, Weuster B, Pfitzer M, Dempfle CE, Stehle G, Kubler W, et al. Prophylaxis of embolic events in patients with atrial fibrillation using low molecular weight heparin. Semin Thromb Hemost. 1993;19(Suppl 1):116-21. | I |
| Hellemons BSP, Langenberg M, Lodder J, Vermeer F, Schouten HJA, Lemmens Th, et al. Primary prevention of arterial thromboembolism in non-rheumatic atrial fibrillation in primary care: randomised controlled trial comparing two intensities of coumarin with aspirin. BMJ 1999;319:958-64. | I |
| Hohnloser SH, Hijazi Z, Thomas L, Alexander JH, Amerena J, et al. (2012) Efficacy of apixaban when compared with warfarin in relation to renal function in patients with atrial fibrillation: insights from the ARISTOTLE trial. Eur Heart J 33: 2821–2830. | P |
| Holmes DR Jr, Kar S, Price MJ, Whisenant B, Sievert H, Doshi SK, Huber K, Reddy VY. Prospective randomized evaluation of the Watchman Left Atrial Appendage Closure device in patients with atrial fibrillation versus long-term warfarin therapy: the PREVAIL trial. J Am Coll Cardiol. 2014;64:1–12. doi: 10.1016/j.jacc.2014.04.029. | I |
| Holmes DR, Reddy VY, Turi ZG, Doshi SK, Sievert H, Buchbinder M, Mullin CM, Sick P; PROTECT AF Investigators. Percutaneous closure of the left atrial appendage versus warfarin therapy for prevention of stroke in patients with atrial fibrillation: a randomised non-inferiority trial. Lancet. 2009;374:534–542. doi: 10.1016/S0140-6736(09)61343-X. | I |
| Hori et al. Efficacy and safety of dabigatran vs. warfarin in patients with atrial fibrillation: Sub-analysis in Japanese population in RE-LY trial. Circ J 2011; 75: 800 – 805 | P |
| Hori M, Matsumoto M, Tanahashi N, Momomura S, Uchiyama S, Goto S, Izumi T, Koretsune Y, Kajikawa M, Kato M, Ueda H, Iwamoto K, Tajiri M; J-ROCKET AF study investigators. Rivaroxaban vs. warfarin in Japanese patients with atrial fibrillation – the J-ROCKET AF study –. Circ J. 2012;76:2104–2111. | P |
| Kakkar AK, Brenner B, Dahl OE, et al. Extended duration rivaroxaban versus short-term enoxaparin for the prevention of venous thromboembolism after total hip arthroplasty: a double-blind, randomised controlled trial. Lancet 2008;372:31–39. | P |
| Lassen MR, Ageno W, Borris LC, et al. Rivaroxaban versus enoxaparin for thromboprophylaxis after total knee arthroplasty. N Engl J Med 2008;358:2776–2786. | P |
| Lassen MR, Davidson BL, Gallus A, et al. The efficacy and safety of apixaban, an oral, direct factor Xa inhibitor, as thromboprophylaxis in patients following total knee replacement. J Thromb Haemost 2007;5:2368–2375. | P |
| Lassen MR, Gallus A, Raskob GE, et al. Apixaban versus enoxaparin for thromboprophylaxis after hip replacement. N Engl J Med 2010;363:2487–2498 | P |
| Lassen MR, Raskob GE, Gallus A, et al. Apixaban or enoxaparin for thromboprophylaxis after knee replacement. N Engl J Med 2009;361:594–604 | P |
| Lassen MR, Raskob GE, Gallus A, et al. Apixaban versus enoxaparin for thromboprophylaxis after knee replacement (ADVANCE-2): a randomised double-blind trial. Lancet 2010;375:807–815. | P |
| Levine MN, Gu C, Liebman HA, et al. A randomized phase II trial of apixaban for the prevention of thromboembolism in patients with metastatic cancer. J Thromb Haemost 2012;10:807–814. | P |
| Lip GY, Larsen TB, Skjoth F, Rasmussen LH. Indirect comparisons of new oral anticoagulant drugs for efficacy and safety when used for stroke prevention in atrial fibrillation. J Am Coll Cardiol 2012;60:738-46 | S |
| Liu X, Huang H, Yu J, et al. Warfarin compared with aspirin for older Chinese patients with stable coronary heart diseases and atrial fibrillation complications. Int J Clin Pharmacol Ther 2014;52:454e459. | I |
| Lopes RD, Alexander JH, Al-Khatib SM, Ansell J, Diaz R, et al. (2012) Apixaban for reduction in stroke and other ThromboemboLic events in atrial fibrillation (ARISTOTLE) trial: design and rationale. Am Heart J 159: 331–339. | S |
| Mant J, Hobbs FD, Fletcher K, et al; BAFTA Investigators; Midland Research Practices Network (MidReC). Warfarin versus aspirin for stroke prevention in an elderly community population with atrial fibrillation (the Birmingham Atrial Fibrillation Treatment of the Aged Study, BAFTA): a randomised controlled trial. Lancet. 2007;370(9586):493-503. | I |
| Mega JL, Braunwald E, Mohanavelu S, et al. Rivaroxaban versus placebo in patients with acute coronary syndromes (ATLAS ACS-TIMI 46): a randomised, double-blind, phase II trial. Lancet 2009;374:29–38. | P |
| Mega JL, Braunwald E, Wiviott SD, et al. Rivaroxaban in patients with a recent acute coronary syndrome. N Engl J Med 2012;366:9–19. | P |
| Morocutti C, Amabile G, Fattapposta F, Nicolosi A, Matteoli S, Trappolini M, et al. Indobufen versus warfarin in the secondary prevention of major vascular events in nonrheumatic atrial fibrillation. SIFA (Studio Italiano Fibrillazione Atriale) Investigators. Stroke. 1997;28:1015-21. | I |
| Ogawa S, Shinohara Y, Kanmuri K. Safety and efficacy of the oral direct factor x a inhibitor apixaban in Japanesepatients with non-valvular atrial fibrillation. The ARISTOTLE-J study. Circ J. 2011;75:1852–1859. | follow-up length < 12 months |
| Oldgren J, Alings M, Darius H, Diener HC, Eikelboom J, et al. (2011) Risks for stroke, bleeding, and death in patients with atrial fibrillation receiving dabigatran or warfarin in relation to the CHADS2 score: a subgroup analysis of the RE-LY trial. Ann Intern Med 155: 660–667, W204 | P |
| Oldgren J, Budaj A, Granger CB, et al. Dabigatran vs. placebo in patients with acute coronary syndromes on dual antiplatelet therapy: a randomized, double-blind, phase II trial. Eur Heart J 2011; 32:2781–2789. | P |
| Pengo V, Zasso A, Barbero F, Banzato A, Nante G, Parissenti L, et al. Effectiveness of fixed minidose warfarin in the prevention of thromboembolism and vascular death in nonrheumatic atrial fibrillation. Am J Cardiol. 1998; 82:433-7. | I |
| Pérez-Gómez F, et al.; NASPEAF Investigators. Comparative effects of antiplatelet, anticoagulant, or combined therapy in patients with valvular and nonvalvular atrial fibrillation: a randomized multicenter study. J Am Coll Cardiol 2004; 44:1557–1566. | I |
| Petersen P, Boysen G, Godtfredsen J, Andersen ED, Andersen B. Placebo-controlled, randomized trial of warfarin and aspirin for prevention of thromboembolic complications in chronic atrial fibrillation: the Copenhagen AFASAK study. Lancet 1989;1:175-9. | I |
| Posada IS, Barriales V. Alternate-day dosing of aspirin in atrial fibrillation. LASAF Pilot Study Group. Am Heart J. 1999;138(1Pt1):137–143. | I |
| Rash A, Downes T, Portner R, et al. A randomised controlled trial of Warfarin versus Aspirin for Stroke Prevention in Octogenarians with atrial fibrillation (WASPO). Age Ageing 2007;36:151–6. | I |
| Raskob G, Cohen AT, Eriksson BI, et al. Oral direct factor Xa inhibition with edoxaban for thromboprophylaxis after elective total hip replacement. A randomised double-blind dose-response study. Thromb Haemost 2010;104:642–649. | P |
| Sato H, Ishikawa K, Kitabatake A, et al. Low-dose aspirin for prevention of stroke in low-risk patients with atrial fibrillation: Japan atrial fibrillation stroke trial. Stroke 2006;37:447–51. | I |
| Schulman S, Kakkar AK, Goldhaber SZ, Schellong S, Eriksson H, Mismetti P, Christiansen AV, Friedman J, Le Maulf F, Peter N, Kearon C; RE-COVER II Trial Investigators. Treatment of acute venous thromboembolism with dabigatran or warfarin and pooled analysis. Circulation. 2014;129:764–772. doi: 10.1161/CIRCULATIONAHA.113.004450 | P |
| Schulman S, Kearon C, Kakkar AK et al. Extended use of dabigatran, warfarin, or placebo in venous thromboembolism. N Engl J Med 2013;368:709–718. | P |
| Schulman S, Kearon C, Kakkar AK, Mismetti P, Schellong S, Eriksson H, Baanstra D, Schnee J, Goldhaber SZ; RE-COVER Study Group. Dabigatran versus warfarin in the treatment of acute venous thromboembolism. N Engl J Med. 2009;361:2342–2352. doi: 10.1056/NEJMoa0906598. | P |
| SPORTIF Executive Steering Committee for the SPORTIF V Investigators. Ximelagatran vs warfarin for stroke prevention in patients with nonvalvular atrial fibrillation: a randomized trial. JAMA 2005;293:690–8. // Albers GW, et al.; for the SPORTIF Executive Steering Committee for the SPORTIF V Investigators. Ximelagatran vs. warfarin for stroke prevention in patients with nonvalvular atrial fibrillation: a randomized trial. J Am Med Assoc 2005; 293:690–698. | I |
| SPORTIF II Investigators. Ximelagatran versus warfarin for stroke prevention in patients with nonvalvular atrial fibrillation. SPORTIF II: a dose-guiding, tolerability, and safety study. J Am Coll Cardiol. 2003;41:1445-51. [PMID:12742279] | I |
| Stroke Prevention in Atrial Fibrillation Investigators. Adjusted-dose warfarin versus low-intensity, fixed-dose warfarin plus aspirin for high-risk patients with atrial fibrillation: Stroke Prevention in Atrial Fibrillation III randomised clinical trial. Lancet 1996;348:633–8. | I |
| Stroke Prevention in Atrial Fibrillation Investigators. Stroke Prevention in Atrial Fibrillation Study. Final results. Circulation 1991;84:527–39. | I |
| Stroke Prevention in Atrial Fibrillation Investigators. Warfarin versus aspirin for prevention of thromboembolism in atrial fibrillation: Stroke Prevention in Atrial Fibrillation II Study. Lancet 1994;343:687–91 | I |
| The SPAF II Investigators. Bleeding during antithrombotic therapy in patients with atrial fibrillation. The Stroke Prevention in Atrial Fibrillation Investigators. Arch Intern Med 1996; 156: 409–416. | I |
| The SPAF III Writing Committee for the Stroke Prevention in Atrial Fibrillation Investigators. Patients with nonvalvular atrial fibrillation at low risk of stroke during treatment with aspirin: stroke prevention in atrial fibrillation III study. JAMA 1998; 279: 1273–7. | S |
| Turpie AG, Bauer KA, Davidson BL, et al. A randomized evaluation of betrixaban, an oral factor Xa inhibitor, for prevention of thromboembolic events after total knee replacement (EXPERT). Thromb Haemost 2009;101:68–76. | P |
| Turpie AG, Fisher WD, Bauer KA, et al. BAY 59-7939: an oral, direct factor Xa inhibitor for the prevention of venous thromboembolism in patients after total knee replacement. A phase II dose-ranging study. J Thromb Haemost 2005;3:2479–2486. | P |
| Turpie AG, Lassen MR, Davidson BL, et al. Rivaroxaban versus enoxaparin for thromboprophylaxis after total knee arthroplasty (RECORD4): a randomised trial. Lancet 2009;373:1673–1680. | P |
| Vemmos KN, Tsivgoulis G, Spengos K, Manios E, Xinos K, Vassilopoulou S, et al. Primary prevention of arterial thromboembolism in the oldest old with atrial fibrillation—a randomized pilot trial comparing adjusted-dose and fixed low-dose coumadin with aspirin. Eur J Intern Med. 2006;17:48-52. [PMID:16378886] | I |
| Wallentin L, Wilcox R, Weaver WD, Emanuelsson H, Goodvin A, Nyström P, Bylock A. Oral ximelagatran for secondary prophylaxis after myocardial infarction: the ESTEEM randomised controlled trial. Lancet 2003;362:789 –797. | P |
| Weitz JI, Connolly SJ, Patel I, et al. Randomised, parallel-group, multicentre, multinational phase 2 study comparing edoxaban, an oral factor Xa inhibitor, with warfarin for stroke prevention in patients with atrial fibrillation. Thromb Haemost 2010;104:633e641. | P |
| Yamaguchi T. Optimal intensity of warfarin therapy for secondary prevention of stroke in patients with nonvalvular atrial fibrillation: a multicenter, prospective, randomized trial. Japanese Nonvalvular Atrial Fibrillation-Embolism Secondary Prevention Cooperative Study Group. Stroke. 2000;31:817-21. [PMID: 10753981] | I |
| Yamashita T, Koretsune Y, Yasaka M, et al. Randomized, multicenter, warfarin-controlled phase II study of edoxaban in Japanese patients with non-valvularatrial fibrillation. Circ J. 2012;76:1840–1847. | P |

**P:** wrong patients; **I:** wrong intervention; **S:** wrong study type **O:** no relevant outcome

**Supplement IV: data extraction sheets**

| **Study/Reference** | **Region, Setting, Main Inclusion Criteria, Main Exclusion Criteria and Baseline Characteristics of Study Population** | **Intervention(s), Control, Patient Flow (IG/CG), Length of Follow-up Period, and Treatment Period** |
| --- | --- | --- |
| Main trial publication: Granger CB, Alexander JH, McMurray JJ, Lopes RD, Hylek EM, Hanna M, Al-Khalidi HR, Ansell J, Atar D, Avezum A, Bahit MC, Diaz R, Easton JD, Ezekowitz JA, Flaker G, Garcia D, Geraldes M, Gersh BJ, Golitsyn S, Goto S, Hermosillo AG, Hohnloser SH, Horowitz J, Mohan P, Jansky P, Lewis BS, Lopez-Sendon JL, Pais P, Parkhomenko A, Verheugt FW, Zhu J, Wallentin L. Apixaban versus warfarin in patients with atrial fibrillation. N Engl J Med 2011;365:981–992. . **(Subgroup analysis Age ≥65 years)**  Halvorsen S, Atar D, Yang H, De Caterina R, Erol C, Garcia D, et al. Efficacy and safety of apixaban compared with warfarin according to age for stroke prevention in atrial fibrillation: Observations from the ARISTOTLE trial. European Heart Journal. 2014;35(28):1864-72. **(Subgroup analysis age ≥ 65 years)**  Alexander JH, Andersson U, Lopes RD, Hijazi Z, Hohnloser SH, Ezekowitz JA, et al. Apixaban 5 mg twice daily and clinical outcomes in patients with atrial fibrillation and advanced age, low body weight, or high creatinine: A secondary analysis of a randomized clinical trial. JAMA Cardiology. 2016;1(6):673-81. **(Subgroup analysis age ≥80 years)** | **Region/Setting**  1034 clinical sites in 39 countries  December 2006 - April 2010  ARISTOTLE, NCT00412984  **Main inclusion criteria**   - AF or flutter at enrollment or two or more episodes of atrial fibrillation or flutter, as documented by electrocardiography, at least 2 weeks apart in the 12 months before enrollment - at least one of the following risk factors for stroke: - an age of at least 75 years - previous stroke - TIA, or systemic embolism - symptomatic heart failure within the previous 3 months or left ventricular ejection fraction of no more than 40% - diabetes mellitus - hypertension requiring pharmacologic treatment   **Main exclusion criteria**   - AF due to a reversible cause - moderate or severe mitral stenosis - conditions other than atrial fibrillation that required anticoagulation (e.g., a prosthetic heart valve) - stroke within the previous 7 days - a need for aspirin at a dose of >165 mg a day or for both aspirin and clopidogrel - severe renal insufficiency (serum creatinine level of >2.5 mg per deciliter [221 μmol per liter] or calculated creatinine clearance of <25 ml per minute)   **Patient characteristics for age category 65 to <75years/≥75years**  Age group n(%)  65 to <75 years 7052(39)  ≥75 years 5678(31)  Gender n(%)  Male 4527(64.2)/3282(57.8)  Female 2525(35.8)/2396(42.2)  Weight [kg] Mean(SD)  84.1(19.2)/76.5(16.4)  HAS-BLED-Score n(%)  1 2008(28.5)/1322(23.3)  2 3078(43.6)/2442(43.0)  ≥3 1966(27.9)/1914(33.7)  Renal function by Cockcroft–Gault, n(%)  Normal (>80 ml/min) 2761(39.2)/597(10.5)  Mild impairment (>50 to 80 ml/min) 3511(49.8)/2922(51.5)  Moderate impairment (>30 to 50 ml/min) 713(10.1)/1906(33.6)  Severe impairment (≤30 ml/min) 40(0.6)/222(3.9)  Co-medication n(%)  Prior use of VKA for >30 consecutive days 4080(57.9)/3390(59.7)  ACE inhibitor or ARB 5198(74.5)/3666(65.7)  Amiodarone 770(11.0)/481(8.6)  Beta-blocker 4573(65.6)/3266(58.5)  Aspirin 2274(32.2)/1729(30.5)  Clopidogrel 135(1.9)/120(2.1)  Digoxin 2211(31.7)/1754(31.4)  Calcium blocker 2296(32.9)/1833(32)  Lipid lowering agents 3346(48.0)/2630(47.1)  Statins 3069(44.0)/2372(42.5)  Nonsteroidal antiinflammatory agent 568(8.1)/631(11.3)  Gastric antacid drugs 1211(17.4)/1400(25.1)  Co-morbidities n(%)  Prior myocardial infarction 1032(14.6)/879(15.5)  Prior bleeding 1185(16.8)/1169(20.6)  History of fall within previous year 254(4.0)/379(7.3)  Prior stroke, TIA, or systemic embolism 1390(19.7)/1238(21.8)  Congestive heart failure 2195(31.1)/1378(24.3)  Diabetes 1935(27.4)/1200(21.1)  Hypertension 6448(91.4)/4715(83.0) | **Intervention**  Apixaban  5 mg twice daily  **Dose reduction**  2.5 mg twice daily for patients with two or more of the following factors: age ≥80 years, bodyweight ≤60 kg, serum creatinine ≥133 μmol/L (≥1.5 mg/dL)  **Control**  Dose-adjusted (INR 2–3) warfarin  **Randomized patients (aged ≥65y) n**  6389/6341  **Follow-up [y] Median(IQR)**  1.8(1.4–2.3)  **Treatment period**  NR |
| Main trial publication:  Connolly SJ, Eikelboom J, Joyner C, et al. AVERROES steering committee and investigators. Apixaban in patients with atrial fibrillation. N Engl J Med 2011;364:806–17.  Ng KH, Shestakovska O, Connolly SJ, Eikelboom JW, Avezum A, Diaz R, Lanas F, Yusuf S, Hart RG. Efficacy and safety of apixaban compared with aspirin in the elderly: a subgroup analysis from the AVERROES trial. Age Ageing. 2016 Jan;45(1):77-83. **(subgroup analysis)** | **Region/Setting**  522 sites in 36 countries  September 2007 - December 2009  AVERROES, NCT00496769  **Main inclusion criteria**   - 50 years of age or older - AF documented in the 6 months before enrollment or by 12-lead electrocardiography on the day of screening - at least one of the following risk factors for stroke: - prior stroke or TIA - age of 75 years or older - arterial hypertension (receiving treatment) - diabetes mellitus (receiving treatment) - heart failure (NYHA class 2 or higher at the time of enrollment) - left ventricular ejection fraction of 35% or less, or documented peripheral-artery disease - patients could not be receiving VKA therapy, either because it had already been demonstrated to be unsuitable for them or because it was expected to be unsuitable. The reasons that VKA therapy was unsuitable for the patient had to be documented on the study case report forms   **Main exclusion criteria**   - presence of conditions other than AF for which the patient required long-term anticoagulation - valvular disease requiring surgery - a serious bleeding event in the previous 6 months or a high risk of bleeding (e.g., active peptic ulcer disease, a platelet count of <100,000/mm^3^ or hemoglobin level of <10 g/dL, stroke within the previous 10 days, documented hemorrhagic tendencies, or blood dyscrasias) - current alcohol or drug abuse or psychosocial issues - life expectancy of less than 1 year - severe renal insufficiency (a serum creatinine level of >2.5 mg/dL [221 μmol/L] or a calculated creatinine clearance of <25 ml/min) - an alanine aminotransferase or aspartate aminotransferase level greater than 2x ULN range or a total bilirubin more than 1.5x ULN range - allergy to aspirin   **Patient characteristics (patients ≥ 75 years) IG/CG**  Age [y] Mean(SD)  80.4(4.3)/80.4(4.2)  Age category n(%)  ≥75 years 1898(33.9)  <74 years 3700(66.1)  Gender n(%)  Male 473(52.2)/506(50.0)  Female 433(47.8)/486(50.0)  Weight Mean(SD)  72.7(16.0)/72.4(14.9)  BMI [kg/m^2^] Mean(SD)  26.9(4.9)/27.0(4.7)  HAS-BLED-Score  NR  Renal function, eGFR Mean(SD)  59.6(16.1)/60.6(16.1)  Renal function, eGFR n(%)  <60 mL/min 466(51.4)/490(49.4)  ≥60 mL/min 440(48.6)/502(50.6)  Co-medication n(%)  Previous VKA use 59.6(16.1)/60.6(16.1)  Study dose of 2.5 mg twice daily of apixaban or apixaban-placebo 169(18.7)/167(16.8)  Co-morbidities n(%)  Prior stroke or TIA 153(16.9)/170(17.2)  Hypertension, receiving treatment 721(79.6)/802(81.0)  Heart failure 336(37.1)/363(36.7)  Diabetes, receiving treatment 168(18.5)/200(20.2) | **Intervention**  Apixaban  5 mg twice daily  **Dose reduction**  2.5 mg twice daily for patients with two or more of the following factors: age ≥80 years, body weight ≤60 kg, or serum creatinine ≥1.5 mg/dl or 133 μmol/l  **Control**  Acetylsalicylic acid  81 - 324 mg daily  **Randomized patients (aged ≥75y) n**  906/992  **Follow-up [y] Mean**  1.1  **Treatment period**  NR |
| Okumura K, Akao M, Yoshida T, et al. Low‐Dose Edoxaban in Very Elderly Patients with Atrial Fibrillation. N Engl J  Med. DOI: 10.1056/NEJMoa2012883. | **Region/Setting**  164 sites in Japan  August 2016 - November 2019  ELDERCARE-AF, NCT02801669  **Main inclusion criteria**   - patients aged ≥80 years - history of nonvalvular atrial fibrillation documented on an electrocardiogram or on a monitor recording obtained within 1 year before consent was given - CHADS_2_ score ≥2 - inappropriate candidates for oral anticoagulants (i.e., warfarin, dabigatran, rivaroxaban, apixaban, or edoxaban) at the recommended therapeutic strength (in the case of warfarin) or at approved doses for one or more of the following reasons: - low CrCl (15-30 ml/min) - history of bleeding from a critical area or organ or gastrointestinal bleeding - low body weight (≤45 kg) - continuous use of nonsteroidal antiinflammatory drugs (NSAIDs) - current use of an antiplatelet drug   **Main exclusion criteria**  NR  **Patient characteristics IG/CG**  Age [y] Mean(SD)  86.7(4.2)/86.4(4.3)  Age group n(%)  ≥75y 492(100.0)/492(100.0)  ≤85y 218(44.3)/229(46.5)  >85y 274(55.7)/263(53.5)  Gender n(%)  Male 212(43.1)/207(42.1)  Female 280(56.9)/285(57.9)  Weight [kg] Mean(SD)  50.6(10.9)/50.6(11.1)  BMI Mean(SD)  22.1(3.6)/22.2(3.8)  HAS-BLED score Mean(SD)  2.3(0.9)/2.4(0.9)  Renal function: CrCl [ml/min] Mean(SD)  36.3(14.3)/36.2(14.5)  Renal function: CrCl n(%)  ≤50 ml/min 415(84.3)/408(82.9)  >50 ml/min 77(15.7)/84(17.1)  Co-medication n(%)  NR  Comorbidities n(%)  Previous stroke or TIA 110(22.4)/126(25.6)  Congestive HF 259(52.6)/274(55.7)  Diabetes mellitus 115(23.4)/110(22.4)  Hypertension 412(83.7)/398(80.9)  Coronary artery disease 130(26.4)/127(25.8)  Dementia 70(14.2)/90(18.3)  History of falling within past year 154(31.3)/186(37.8) | **Intervention**  Edoxaban, 15 mg, once daily  **Dose reduction**  NR  **Control**  Placebo  **Randomized patients IG/CG n**  492/492  **Follow-up [days] Median(IQR)**  466.0 (293.5-708.0)  **Treatment period**  NR |
| Main trial publication  Giugliano RP, Ruff CT, Braunwald E, Murphy SA, Wiviott SD, Halperin JL, Waldo AL, Ezekowitz MD, Weitz JI, Špinar J, Ruzyllo W, Ruda M, Koretsune Y, Betcher J, Shi M, Grip LT, Patel SP, Patel I, Hanyok JJ, Mercuri M, Antman EM; ENGAGE AF-TIMI 48 Investigators. Edoxaban versus warfarin in patients with atrial fibrillation. N Engl J Med. 2013;369:2093–2104.  Kato ET, Giugliano RP, Ruff CT, Koretsune Y, Yamashita T, Kiss RG, Nordio F, Murphy SA, Kimura T, Jin J, Lanz H, Mercuri M, Braunwald E, Antman EM. Efficacy and Safety of Edoxaban in Elderly Patients With Atrial Fibrillation in the ENGAGE AF-TIMI 48 Trial. J Am Heart Assoc. 2016 May 20;5(5). | **Region/Setting**  1393 sites in 46 countries  November 2008 - November 2010  ENGAGE AF-TIMI 48, NCT 00781391  **Main inclusion criteria**   - 21 years of age or older - AF documented by means of an electrical tracing within the 12 months preceding randomization - score of 2 or higher on the CHADS_2_ risk assessment - anticoagulation therapy planned for the duration of the trial   **Main exclusion criteria**   - AF due to a reversible disorder - estimated creatinine clearance of less than 30 ml/min - high risk of bleeding - use of dual antiplatelet therapy - moderate-to-severe mitral stenosis - other indications for anticoagulation therapy - acute coronary syndromes - coronary revascularization, or stroke within 30 days before randomization - inability to adhere to study procedures   **Patient characteristics by age category 65 - 74y/** **≥75y**  Age [y] Median(IQR)  70(67.0–72.0)/79(76.0–82.0)  Age category n(%)  ≥75 years 8474(40.2)  65 to 74 years 7134(33.8)  Gender n(%)  Male 4381(61)/4697(55)  Female 2753(39)/3777(45)  Weight Median(IQR)  83.0(71.0–95.7)/76.0(65.9–86.5)  HAS-BLED-Score ≥3 n(%)  4129(58)/4779(56)  Renal function; CrCl (mL/min) at randomization Median(IQR)  74(60–90)/56(45–69)  Renal function [mL/min) at randomization] n(%)  ≤50 813(11)/3116(37)  >50 to 80 3504(49)/4381(52)  >80 2817(40)/977(12)  Co-medication n(%)  Prior VKA experience 4285 (60) 5178 (61)  Aspirin 2073(29)/2460(29)  Thienopyridine 169(2.4)/205(2.4)  Co-morbidities n(%)  Hypertension 6731(94)/7857(93)  Dyslipidemia 3836(54)/4419(52)  Prior stroke or TIA 2334(33)/2119(25)  Diabetes 3053(43)/2336(28)  Prior PCI 500(7.0)/654 (7.7)  Congestive heart failure 4477(63)/3815(45) | **Intervention 1**  Edoxaban  60 mg once-daily  **Intervention 2**  Edoxaban  30 mg once-daily  **Dose reduction**  The dose of edoxaban was reduced by 50% if any of the following factors were present at the time of randomization or during the study: calculated CrCl of ≤50 mL/min using the Cockcroft-Gault formula, body weight ≤60 kg, or the concomitant use of potent P-glycoprotein inhibitors.  **Control**  Dose-adjusted (INR 2–3) warfarin  **Randomized patients (aged 65 to 74y) n**  7134  **Randomized patients (aged ≥75y) n**  8474  **Follow-up [y] Median**  2.8  **Treatment period [d] Median**  **907** |
| Main trial publication:  Connolly SJ, Ezekowitz MD, Yusuf S, Eikelboom J, Oldgren J, Parekh A, et al; RE-LY Steering Committee and Investigators. Dabigatran versus warfarin in patients with atrial fibrillation. N Engl J Med. 2009;361:1139-51.  Eikelboom JW, Wallentin L, Connolly SJ, Ezekowitz M, Healey JS, et al. (2011) Risk of bleeding with 2 doses of dabigatran compared with warfarin in older and younger patients with atrial fibrillation: an analysis of the randomized evaluation of long-term anticoagulant therapy (RE-LY) trial. Circulation 123: 2363–2372  Lauw MN, Eikelboom JW, Coppens M, Wallentin L, Yusuf S, Ezekowitz M, et al. Effects of dabigatran according to age in atrial fibrillation. Heart. 2017;103(13):1015-23 | **Region/Setting**  951 sites in 44 countries  December 2005 - December 2007  RE-LY, NCT00262600  **Main inclusion criteria**   - AF documented on electrocardiography performed at screening or within 6 months beforehand - at least one of the following characteristics: - previous stroke or TIA - left ventricular ejection fraction of less than 40% - NYHA class II or higher heart-failure symptoms within 6 months before screening - age of at least 75 years or an age of 65 to 74 years plus diabetes mellitus, hypertension, or coronary artery disease   **Main exclusion criteria**   - presence of a severe heart-valve disorder - stroke within 14 days or severe stroke within 6 months before screening - condition that increased the risk of hemorrhage - creatinine clearance of less than 30 ml/min - active liver disease - pregnancy   **Patient characteristics IG1/IG2/CG (all patients)**  Age [y] Mean(SD)  71.4(8.6)/71.5(8.8)/71.6(8.6)  Gender n/N(%)  Male 3865/6015(64.3)/3840/6076(63.2)/3809/6022(63.3)  Female 2150(35.7)/2236(36.8)/2213(36.7)  Weight [kg] Mean(SD)  82.9(19.9)/82.5(19.4)/82.7(19.7)  HAS-BLED-Score  NR  Renal function  NR  Co-medication n(%)  Aspirin 2404(40.0)/2352(38.7)/2442(40.6)  ARB or ACE inhibitor 3987(66.3)/4053(66.7)/3939(65.5)  Beta-blocker 3784(62.9)/3872(63.7)/3719(61.8)  Amiodarone 624(10.4)/665(10.9)/644(10.7)  Statin 2698(44.9)/2667(43.9)/2673(44.4)  Proton-pump inhibitor 812(13.5)/847(13.9)/832(13.8)  H2-receptor antagonist 225(3.7)/241(4.0)/256(4.3)  Long-term VKA therapy 3011(50.1)/3049(50.2)/2929(48.6)  Co-morbidities n(%)  Previous stroke or TIA 1195(19.9)/1233(20.3)/1195(19.8)  Prior myocardial infarction 1008(16.8)/1029(16.9)/968(16.1)  Heart failure 1937(32.2)/1934(31.8)/1922(31.9)  Diabetes mellitus 1409(23.4)/1402(23.1)/1410(23.4)  Hypertension 4738(78.8)/4795(78.9)/4750(78.9) | **Intervention 1**  Dabigatran  110 mg twice daily  **Intervention 2**  Dabigatran  150 mg twice daily  **Dose reduction**  NR  **Control**  Dose-adjusted (INR 2–3) warfarin  **Randomized patients (aged ≥65y) n**  5017/5046/5069  **Follow-up [y] Median**  2.0  **Treatment period**  NR |
| Main trial publication:  Patel MR, Mahaffey KW, Garg J, Pan G, Singer DE, Hacke W, et al; ROCKET AF Investigators. Rivaroxaban versus warfarin in nonvalvular atrial fibrillation. N Engl J Med. 2011;365:883-91.  Halperin JL, Hankey GJ, Wojdyla DM, et al. Efficacy and safety of rivaroxaban compared with warfarin among elderly patients with nonvalvular atrial fibrillation in the rivaroxaban once-daily, oral, direct factor xa inhibition compared with vitamin k antagonism for prevention of stroke and embolism trial in atrial fibrillation (ROCKET AF). Circulation 2014;130:138e146.**(Subgroup analysis patients aged ≥75)** | **Region/Setting**  1178 sites in 45 countries  December 2006 - June 2009  ROCKET-AF, NCT00403767  **Main inclusion criteria**   - NVAF as documented on electrocardiography - moderate-to-high risk for stroke. Elevated risk was indicated by a history of stroke, TIA, or systemic embolism or at least two of the following risk factors: - heart failure - left ventricular ejection fraction of 35% or less - hypertension - age of 75 years or more - presence of diabetes mellitus (i.e., a CHADS_2_ score of 2 or more, on a scale ranging from 1 to 6, with higher scores indicating a greater risk of stroke)   **Main exclusion criteria**   - cardiac-related conditions - Hemorrhage risk-related criteria - concomitant conditions and therapies   **Patient characteristics (patients ≥ 75 years) IG/CG**  Age [y] Median(25th, 75th percentiles)  79(76, 82)/79(76, 82)  Gender n(%)  Male 1674(53.6)/1677(53.9)  Female 1446(46.4)/1432(46.1)  BMI [kg/m^2^] Median((25th, 75th percentiles)  27.4(24.7, 30.7)/27.2(24.6, 30.4)  HAS-BLED-Score  NR  Renal function, creatinine clearance [ml/min] Median(25th, 75th percentiles)  55(44, 68)/55(44, 68)  Co-medication n(%)  Prior aspirin use 1076(34.5)/1074(34.5)  Prior VKA use 2055(65.9)/2056(66.1)  Co-morbidities n(%)  Prior TIA/stroke or systemic embolism 1297(41.6)/1295(41.7)  Congestive heart failure 1839(58.9)/1811(58.3)  Hypertension 2877(92.2)/2900(93.3)  Diabetes mellitus 1071(34.3)/1031(33.2)  Prior MI 543(17.4)/609(19.6)  PVD 197(23.5)/206(24.6)  COPD 413(27.6)/368(24.6) | **Intervention**  Fixed-dose rivaroxaban 20 mg daily  **Dose reduction**  15 mg daily in patients with a CrCl of 30 to 49 ml/min  **Control**  Dose-adjusted (INR 2–3) warfarin  **Randomized patients (aged ≥75y) n**  3120/3109  **Follow-up [d] Median**  707 **Treatment period [d] Median**  590 |
| Main trial publication:  Cannon CP, Bhatt DL, Oldgren J, Lip GYH, Ellis SG, Kimura T, et al. Dual antithrombotic therapy with dabigatran after PCI in atrial fibrillation. New England Journal of Medicine. 2017;377(16):1513-24.  ten Berg JM, Steg PG, Bhatt DL, Hohnloser SH, de Veer A, Nordaby M, et al. Comparison of the Effect of Age (< 75 Versus ≥ 75) on the Efficacy and Safety of Dual Therapy (Dabigatran+Clopidogrel or Ticagrelor) Versus Triple Therapy (Warfarin+Aspirin+Clopidogrel or Ticagrelor) in Patients With Atrial Fibrillation After Percutaneous Coronary Intervention (from the RE-DUAL PCI Trial). American Journal of Cardiology. 2020;125(5):735-43.**(subgroup analysis)** | **Region/Setting**  414 sites in 41 countries  July 2014 - October 2016  RE-DUAL PCI, NCT02164864  **Main inclusion criteria**   - ≥18 years of age (age subgroup analysis) - NVAF and had successfully undergone PCI with a bare-metal or drug-eluting stent within the previous 120 hours - NVAF could be paroxysmal, persistent, or permanent, but it could not be secondary to a reversible disorder unless long-term treatment with an oral anticoagulant was anticipated - patients treated and those not treated with an oral anticoagulant before PCI - indication for PCI could be either an acute coronary syndrome or stable coronary-artery disease   **Main exclusion criteria**   - presence of bioprosthetic or mechanical heart valves, severe renal insufficiency (creatinine clearance, <30 ml/min), or other major coexisting conditions   **Patient characteristics (patients aged ≥75 years)**  Age [y] Mean(SD)  79.3(3.6)  Gender n(%)  Male 704(68.6)  Female 322(31.4)  Weight / BMI [kg/m^2^]  NR  Modified HAS-BLED-Score Mean(SD)  3.0(0.6)  Modified HAS-BLED-Score n(%)  <3 151(14.7)  ≥ 3 875(85.3)  Co-medication n(%)  NR  Co-morbidities n(%)  Diabetes mellitus 343(33.4)  Previous stroke 103(10.0)  Previous PCI 337(32.8)  Previous stent thrombosis 24(2.3)  Previous coronary artery disease 692(67.4)  Previous myocardial infarction 247(24.1)  Previous CABG 117(11.4)  Previous pulmonary embolism 16(1.6)  Previous systemic embolism 7(0.7) | **Intervention 1**  **Dabigatran 110 mg dual:**  Dabigatran etexilate (110 mg twice daily) plus either clopidogrel or ticagrelor  **Intervention 2**  **Dabigatran 150 mg dual:**  Dabigatran etexilate (150 mg twice daily) plus either clopidogrel or ticagrelor  **Dose reduction**  Elderly patients in non-US countries (≥ 80 years) or Japan (≥ 70 years) were randomized to receive dabigatran 110 mg dual or warfarin triple therapy, whereas younger patients in non-US countries (< 80 years) and Japan (< 70 years), and all US patients irrespective of age, were randomized to dabigatran 110 mg dual, dabigatran 150 mg dual, or warfarin triple therapy.  **Control**  **Warfarin triple therapy:**  Warfarin + aspirin (≤100 mg daily, 1 month when a bare-metal stent was used, and for 3 months when a drug-eluting stent was used) + either clopidogrel or ticagrelor  **Randomized patients (aged ≥75y) n**  1026  **Follow-up Mean**  14.0 months  **Treatment period Mean**  12.3 months |
| Gibson CM, Mehran R, Bode C, Halperin J, Verheugt FW, Wildgoose P, et al. Prevention of bleeding in patients with atrial fibrillation undergoing PCI. New England Journal of Medicine. 2016;375(25):2423-34. | **Region/Setting**  431 sites  May 2013 - July 2015  PIONEER AF PCI, NCT01830543  **Main inclusion criteria**   - at least 18 years of age - patients just undergone PCI with stent placement - documented paroxysmal, persistent, or permanent nonvalvular atrial fibrillation (defined as atrial fibrillation not considered to be caused by a primary valve stenosis) that occurred within 1 year before screening; patients with documented atrial fibrillation that occurred more than 1 year before screening were also eligible if the participant had been receiving oral anticoagulation for atrial fibrillation for the 3 months immediately preceding the index PCI   **Main exclusion criteria**   - history of stroke or TIA - clinically significant gastrointestinal bleeding within 12 months before randomization - calculated creatinine clearance of less than 30 ml/min - anemia of an unknown cause with a hemoglobin concentration of less than 10 g/dl - any other condition known to increase the risk of bleeding   **Patient characteristics IG/CG (all patients)**  Age [y] Mean(SD)  70.0(9.1)/69.9(8.7)  Age group n(%)  ≥65 y of age 523(73.8)/516(72.8)/526(74.5)  ≥75 y of age 254(35.8)/245(34.6)/230(32.6)  Gender n(%)  Male 535(75.5)/518(73.4)  Female 174(24.5)/188(26.6)  BMI [kg/m^2^] Median(IQR)  28.4(25.6 – 32.1)/29.0(25.8 – 32.8)  HAS-BLED-Score n(%)  0 2(0.3)/0(0.0)  1 43(6.1)/26(3.7)  2 182(25.7)/182(25.8)  3 294(41.5)/308(43.6)  4 157(22.1)/157(22.2)  5 30(4.2)/31(4.4)  6 1(0.1)/2(0.3)  Renal function, Creatinine clearance [ml/min] Mean(SD)  77.5(31.8)/80.7(30.0)  Co-medication n(%)  Clopidogrel 664(93.7)/680(96.3)  Prasugrel 11(1.6)/5(0.7)  Ticagrelor 34(4.8)/21(3.0)  Aspirin 702(99.7)/699(99.6)  Beta-blocker 541(76.3)/537(76.1)  ACE inhibitor or ARB 532(75.0)/537(76.1)  Statin 557(78.6)/552(78.2)  PPI: Omeprazole or esomeprazole 78(11.0)/79(11.2)  PPI: Other 198(27.9)/180(25.5)  Co-morbidities  Congestive heart failure 187(26.4)/175(24.8)  Hypertension 519(73.2)/532(75.4)  Diabetes mellitus 199(28.1)/221(31.3)  Hypercholesterolemia 295(41.6)/316(44.8)  Previous myocardial infarction 180(25.4)/157(22.2)  Peripheral vascular disease 42(5.9)/35(5.0)  Gastrointestinal bleeding 9(1.3)/5(0.7) | **Intervention**  Rivaroxaban, 2.5 mg twice daily plus background DAPT(low-dose aspirin (75 to 100 mg per day) and clopidogrel, 75 mg once daily (or ticagrelor, 90 mg twice daily or prasugrel,10 mg once daily in ≤15% of participants) for a prespecified duration of 1, 6, or 12 months  Participants who received the treatment for 1 or 6 months then received rivaroxaban, 15 mg once daily (or 10 mg once daily if they had moderate renal impairment) plus background single antiplatelet therapy with low-dose aspirin (75 to 100 mg per day) for the remainder of the 12-month treatment period  **Control**  Warfarin once daily (dose adjustment to achieve a target INR of 2.0 to 3.0) plus background DAPT with low-dose aspirin (75 to 100 mg per day) and clopidogrel, 75 mg once daily (or ticagrelor, 90 mg twice daily or prasugrel, 10 mg once daily in ≤15% of participants) for a prespecified duration of 1, 6, or 12 months  Participants who received the treatment for 1 or 6 months then received warfarin once daily (with dose adjustment to achieve a target INR of 2.0 to 3.0) plus background single antiplatelet therapy with low-dose aspirin (75 to 100 mg per day) for the remainder of the 12-month treatment period  **Randomized patients (≥65 y) n**  516/526  **Randomized patients (≥75 y) n**  254/245/230  **Follow-up**  NR  **Treatment period**  12 months |
| Vranckx P, Valgimigli M, Eckardt L, Tijssen J, Lewalter T, Gargiulo G, et al. Edoxaban-based versus vitamin K antagonist-based antithrombotic regimen after successful coronary stenting in patients with atrial fibrillation (ENTRUST-AF PCI): a randomised, open-label, phase 3b trial. The Lancet. 2019;394(10206):1335-43. | **Region/Setting**  186 sites in 18 countries  February 2017 - May 2018  ENTRUST-AF PCI, NCT02866175  **Main inclusion criteria**   - aged at least 18 years (age subgroup analysis) - AF requiring oral anticoagulation and non-valvular AF not secondary to a reversible disorder - successful PCI for stable coronary artery disease or acute coronary syndrome   **Main exclusion criteria**   - patients with mechanical heart valves - moderate-to-severe mitral stenosis - end-stage renal disease - other major comorbidities   **Patient characteristics IG/CG (all patients)**  Age [y] Median(IQR)  69(63-77)/70(64-77)  Gender n(%)  Male 557(74)/563(75)  Female 194(26)/192(25)  Weight [kg] Median(IQR)  80(71-93)/83(72-94)  HAS-BLED-Score Median(IQR)  3.0(2.0-3.0)/3.0(2.0-3.0)  Renal function, creatinine clearance [mL/min], Median(IQR)  71.8(53.7-91.1)/71.7(54.0-90.9)  Co-medication n(%)  VKA 232(31)/224(30)  NOAC 176(23)/189(25)  None 192(26)/221(29)  Data missing 151(20)/121(16)  Clopidogrel 696(93)/695(92)  Prasugrel 5 mg 2(<1)/1(<1)  Prasugrel 10 mg 3(<1)/2(<1)  Ticagrelor 49(7)/57(8)  Co-morbidities n(%)  Acute coronary syndrome 388(52)/389(52)  Stable coronary artery disease 363(48)/366(48)  Myocardial infarction 188(25)/177(23)  Previous PCI 199(26)/195(26)  Previous CABG 46(6)/49(6)  Congestive heart failure 418(56)/408(54)  Stroke 97(13)/92(12)  Peripheral artery disease 76(10)/82(11)  Non-CNS systemic embolic event 12(2)/10(1)  Diabetes mellitus 259(34)/258(34)  Hypertension 674(90)/687(91)  Hypercholesterolaemia 497(66)/484(64)  Bleeding events 56(7)/49(6)  Valvular heart disease 210(28)/221(29)  Malignancy 43(6)/46(6) | **Intervention**  Edoxaban  60 mg once-daily plus clopidogrel, 75 mg once-daily (or prasugrel,5 mg or 10 mg once daily or ticagrelor, 90 mg twice daily)  **Dose reduction**  Edoxaban 30 mg once daily (for patients with any of the following characteristics: moderate or severe renal impairment (calculated creatinine clearance 15–50 mL/min), bodyweight ≤60 kg, or concurrent use of specific potent P-glycoprotein inhibitors (cyclosporine, dronedarone, erythromycin, or ketoconazole)  **Control**  Dose-adjusted (INR 2.0-3.0) VKA plus clopidogrel, 75 mg once daily (or prasugrel, 5 mg or 10 mg once daily or ticagrelor, 90 mg twice daily) for 12 months and aspirin (100 mg once daily) for a minimum of 1 month and up to 12 months  **Randomized patients (≥ 65y) n**  525/553  **Follow-up [d] Median(IQR)**  364(361–368)  **Treatment period**  12 months |
| de Vriese AS, Caluwé R, Pyfferoen L, de Bacquer D, de Boeck K, Delanote J, et al. Multicenter randomized controlled trial of Vitamin K antagonist replacement by rivaroxaban with or without vitamin K2 in hemodialysis patients with atrial fibrillation: The Valkyrie study. Journal of the American Society of Nephrology. 2020;31(1):186-96. | **Region/Setting**  three sites (AZ Sint-Jan Brugge, Onze Lieve Vrouw Ziekenhuis Aalst, ZNA Middelheim Antwerpen), Belgium  February 2015 - July 2017  Valkyrie study, NCT02610933  **Main inclusion criteria**   - age ≥18 years - end-stage renal disease treated with chronic hemodialysis thrice weekly - NVAF documented by electrocardiogram: paroxysmal (selfterminating) or permanent (accepted by the patient and physician); first-diagnosed (not diagnosed before, irrespective of the duration, of the arrhythmia or related symptoms), new-onset or pre-existing - CHA_2_DS_2_-VASc Score ≥2 - treatment with a VKA or no treatment - female subjects should be postmenopausal, surgically sterilized or willing to use highly effective contraception during the study period   **Main exclusion criteria**   - known intestinal malabsorption or inability to take oral medication - inability to stop co-medication that causes major interactions with rivaroxaban (e.g. ketoconazole, itraconazole, voriconazole, posaconazole, ritonavir, rifampicin, phenytoin, carbamazepine, phenobarbital or St John’s wort) - investigator’s assessment that the subject’s life expectancy is less than 1 year - prosthetic mechanical heart valve - contraindication for anticoagulation - liver dysfunction Child-Pugh grade B-C - pregnancy, breastfeeding, inadequate contraception - inability to understand the nature, scope and possible consequences of the study - incompliance with medication and scheduled investigations, unlikelihood of completing the study   **Patient characteristics IG/CG**  Age [y] Median(IQR)  79.9(74.4-83.9)/80.3(71.5-84.3)  Gender n(%)  Male 35(76.1)/25(56.8)  Female 11(23.9)/19(43.2)  BMI [kg/m^2^] Median (IQR)  24.7(22.0-27.5)/25.6(22.3–30.4)  HAS-BLED-Score Mean(SD)  4.6(0.8)/4.7(0.9)  HAS-BLED-Score Median(IQR)  4(4-5)/5(4-5)  Renal function  end-stage renal disease treated with chronic hemodialysis thrice weekly  Co-medication n(%)  VKA vintage [y] Median(IQR) 1.1(0.0-2.8)/0.9(0.1-4.7)  VKA naïve (<3 months) 17(37.8)/14(31.8)  Pacemaker 11(23.9)/8(18.2)  Co-morbidities n(%)  History of stroke 15(32.6)/16(36.4)  History of acute myocardial infarction 21(45.7)/21(47.7)  History of gastrointestinal bleeding 9(19.6)/12(27.3)  Diabetes 20(43.5)/20(45.5)  Congestive heart failure 17(37.0)/9(20.5)  Preexisting vascular disease 25(54.3)/28(63.6)  Dialysis vintage [y] Median (IQR) 2.7(0.9-5.9)/1.8(0.4-5.5)  Incident dialysis (<3 months) 4(8.7)/9(20.5) | **Intervention**  Rivaroxaban  10 mg daily  **Control**  Adjusted dose (INR 2-3) VKA  **Randomized patients**  46/44  **Follow-up**  18 months  **Treatment period**  18 months |

Abbreviations: ACE: angiotensin-converting enzyme; AF: atrial fibrillation; ARB: angiotensin-receptor blocker; ASA: acetylsalicylic acid; BMI: Body Mass Index; CABG: coronary artery bypass grafting; CHADS_2_: scoring system used to identify patients in need of anticoagulation (congestive heart failure, hypertension, age, diabetes, previous stroke); CHA_2_DS_2_-VASc score reflects the risk of stroke, with values ranging from 0 to 9 and higher scores indicating greater risk d: days; eg: for example; CNS: central nervous system; COPD: chronic obstructive pulmonary disease; CrCl: Creatine Clearance; DAPT: dual antiplatelet therapy; eGFR: estimated glomerular filtration rate; HAS-BLED score reflects the risk of major bleeding in patients with atrial fibrillation who are receiving anticoagulant therapy, with values ranging from 0 to 9 and with higher scores indicating greater risk; INR: international normalized ratio; IQR: interquartile range; n: number; MI: myocardial infarction; NR: not reported; NYHA: New York Heart Association; PCI: percutaneous coronary intervention; PPI: Proton pump inhibitor; PVD: peripheral vascular disease; SD: standard deviation; TIA: transient ischemic attack; ULN: upper limit of normal; VKA: vitamin K antagonist; y: years

**Supplement V: results of studies not include in any meta-analysis**

| ***Low-Dose Edoxaban compared to Placebo, ELDERCARE (n=984)*** |
| --- |
| Major bleeding  HR 1.87 (0.90 to 3.89) |
| Discontinuation due to adverse events  RR 1.024 (0.666 to 1.575) |
| ***Apixaban compared to Aspirin, AVERROES (n=*** ***1898)*** |
| Major bleeding  HR 1.21 (0.69 to 2.12) |
| ***Rivaroxaban compared to VKA in end-stage renal diseases patients, Valkyrie (n=90)*** |
| Morality  RR 0.815 (0.457 to 1.454)  Major Bleeding  RR 0.579 (0.250 to 1.341) |

**Supplemental figure I: Hospitalization Apixaban (N=14.628)**


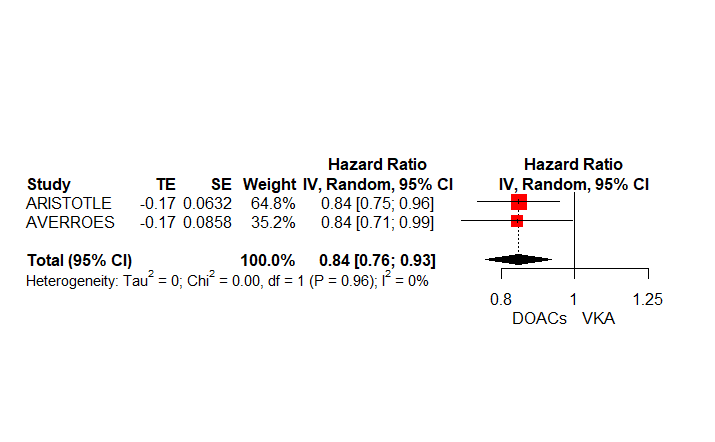

Supplement: Supplementary file 1 — Supplementary file1 (DOCX 96 KB) [file 11357_2023_825_MOESM1_ESM.docx]
